# The *Harbinger* transposon‐derived gene *PANDA* epigenetically coordinates panicle number and grain size in rice

**DOI:** 10.1111/pbi.13799

**Published:** 2022-03-16

**Authors:** Donghai Mao, Shentong Tao, Xin Li, Dongying Gao, Mingfeng Tang, Chengbing Liu, Dan Wu, Liangli Bai, Zhankun He, Xiaodong Wang, Lei Yang, Yuxing Zhu, Dechun Zhang, Wenli Zhang, Caiyan Chen

**Affiliations:** ^1^ Key Laboratory of Agro‐Ecological Processes in Subtropical Region Institute of Subtropical Agriculture Chinese Academy of Sciences Changsha China; ^2^ 70578 State Key Laboratory for Crop Genetics and Germplasm Enhancement Collaborative Innovation Center for Modern Crop Production co‐sponsored by Province and Ministry (CIC‐MCP) Nanjing Agricultural University Nanjing China; ^3^ Small Grains and Potato Germplasm Research Unit USDA ARS Aberdeen ID USA; ^4^ 26476 Key Laboratory of Three Gorges Regional Plant Genetics and Germplasm Enhancement (CTGU)/Biotechnology Research Center China Three Gorges University Yichang China; ^5^ University of Chinese Academy of Sciences Beijing China; ^6^ College of Life Sciences Hunan Normal University Changsha China; ^7^ College of Agronomy Hunan Agriculture University Changsha China; ^8^ Longping Branch Graduate School of Hunan University Changsha China

**Keywords:** rice, transposon‐derived gene, polycomb repressive complex 2, epigenetic regulation, grain yield

## Abstract

Transposons significantly contribute to genome fractions in many plants. Although numerous transposon‐related mutations have been identified, the evidence regarding transposon‐derived genes regulating crop yield and other agronomic traits is very limited. In this study, we characterized a rice *Harbinger* transposon‐derived gene called *PANICLE NUMBER AND GRAIN SIZE* (*PANDA*), which epigenetically coordinates panicle number and grain size. Mutation of *PANDA* caused reduced panicle number but increased grain size in rice, while transgenic plants overexpressing this gene showed the opposite phenotypic change. The *PANDA*‐encoding protein can bind to the core polycomb repressive complex 2 (PRC2) components OsMSI1 and OsFIE2, and regulates the deposition of H3K27me3 in the target genes, thereby epigenetically repressing their expression. Among the target genes, both *OsMADS55* and *OsEMF1* were negative regulators of panicle number but positive regulators of grain size, partly explaining the involvement of *PANDA* in balancing panicle number and grain size. Moreover, moderate overexpression of *PANDA* driven by its own promoter in the *indica* rice cultivar can increase grain yield. Thus, our findings present a novel insight into the epigenetic control of rice yield traits by a *Harbinger* transposon‐derived gene and provide its potential application for rice yield improvement.

## Introduction

Rice (*Oryza sativa*) is a staple food feeding more than 50% of the world’s population. Grain yield in rice is primarily determined by three components including panicle number, grain number and grain weight. A number of genes determining these traits separately have been well characterized and make it possible to improve one of the traits without compromising the other two (Li *et al*., 2018; Wang *et al*., 2018). These traits are usually negatively associated with each other (Sadras, 2007). Recently, more evidence suggests that genetic factors play roles in explaining the negative associations among yield components in plants. For example, *GSN1* coordinates the trade‐off between grain number and grain size by integrating localized cell differentiation and proliferation (Guo *et al*., 2018). *OsSHI1* can interact with *IPA1* and modulates the transcriptional activity of two downstream genes, *OsTB1* and *OsDEP1*, to coordinate panicle number and grain number (Duan *et al*., 2019). A fine gene network composed of microRNAs and transcription factors coordinates rice tiller formation and panicle branching (Wang *et al*., 2015). In addition to the extensively studied balance between panicle/tiller number and grain number, and between grain number and grain size, the balances between panicle number and grain size were also observed (Bai *et al*., 2017; Fu *et al*., 2010; Guo *et al*., 2018). However, the genetic mechanism underlying the coordination between panicle number and grain size is largely unknown.

Polycomb repressive complex 2 (PRC2) epigenetically represses gene expression by catalysing the trimethylation of lysine 27 on histone H3 (H3K27me3) (Bieluszewski *et al*., 2021). It was first discovered in *Drosophila* and then found to be functionally conserved in higher eukaryotes during normal growth and development and in response to environmental cues (Bieluszewski *et al*., 2021). The PRC2 complex consists of four canonical core components and large repertoires of accessory proteins (Bieluszewski *et al*., 2021). The core components are generally not DNA binding proteins, whereas some accessory proteins play roles in sequence‐specific recruitment of PRC2, which defines a selective and flexible target repression in association with the developmental plasticity and dynamics (Kassis and Brown, 2013; Qüesta *et al*., 2016; Xiao *et al*., 2017; Yuan *et al*., 2016, 2021; Zhou *et al*., 2017; Zhou *et al*., 2018). In rice, the paralog pairs OsCLF/OsSET1, OsEMF2a/b, OsFIE1/2 and OsMSI1/2, respectively correspond to the homologous counterparts of the core PRC2 components E(z), Su(z)12, ESC and p55 in *Drosophila* (Liu *et al*., 2014; Luo *et al*., 2009). A number of accessory proteins such as LC2, OsVIL2 and OsEMF1/DS1 modulate PRC2 recruitment by interacting with the core components of PRC2 (Calonje *et al*., 2008; Liu *et al*., 2018; Wang *et al*., 2013; Yang *et al*., 2013). The PRC2 complex has been extensively characterized in various organisms such as *Drosophila*, *Arabidopsis* and mammals, but it is still understudied in rice.

It has been documented that rice panicle number and grain size can be epigenetically controlled by the PRC2 complex. *FIE1* negatively regulates grain width in response to heat stress (Dhatt *et al*., 2021). OsVIL2‐PRC2 suppresses *OsTB1* expression by modifying the chromatin (Wang *et al*., 2013; Yang *et al*., 2013; Yoon *et al*., 2019). NGR5 drives the recruitment of PRC2 to repress the expression of *D14* and *OsSPL14* by interacting with LC2/OsVIL3, thereby subsequently promoting nitrogen‐induced tillering (Wu *et al*., 2020). RLB (RICE LATERAL BRANCH) physically binds to the PRC2 component OsEMF2b to regulate lateral branching through repressing the expression of *OsCKX4* (Wang *et al*., 2021). These studies pinpoint a deep insight regarding a separate mode of regulation of panicle number or grain size by PRC2. However, it is still unknown whether PRC2 can synergistically regulate panicle number and grain size through common regulators in rice. In this study, we identified a rice epigenetic regulator, *PANICLE NUMBER AND GRAIN SIZE*, which fine‐tuned the balance between panicle number and grain size. The gene was identified to be a transposon‐derived gene with neofunctionalization, so we named it ‘*PANDA*’ (*PANICLE NUMBER AND GRAIN SIZE*), to indicate its nature as a living fossil such as the Giant panda (*Ailuropoda melanoleuca*). Mutation of *PANDA* reduced panicle number but increased grain size, while its overexpression plants reversed this balance. More importantly, we found that *PANDA* was neofunctionalized as an epigenetic regulator with an ability to bind PRC2, and the moderate overexpression of *PANDA* driven by its own native promoter can increase grain yield in *indica* rice cultivar.

## Results

### *PANDA* coordinates panicle number and grain size in rice

**Figure 1:**
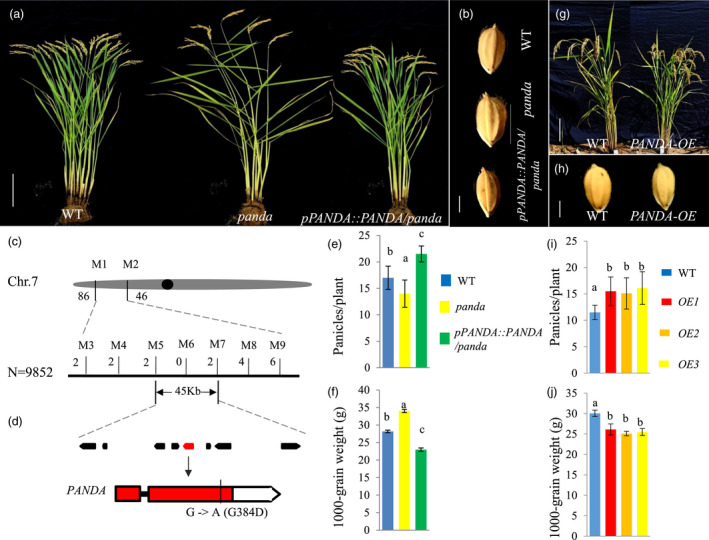
Map‐based cloning of *PANDA*. (a, b) Panicle numbers (a) and grain size (b) in wild type, the mutant *panda* and the complementary line (*pPANDA*::*PANDA/panda*) at maturity. (c) Fine mapping of *PANDA* to a 45‐kb region on the short arm of chromosome 7. (d) Sequence comparison of the candidate gene between wild type and *panda*. (e, f) Statistical analyses of panicle number (e) and grain size (f) in wild type, *panda* and *pPANDA*::*PANDA/panda* plants. (g, h) Panicle number (g) and grain size (h) in wild type and the *PANDA*‐overexpressing (OE) line at maturity. (i, j) Statistical analysis of panicle number (i) and grain size (j) in the wild type and three *PANDA*‐overexpressing lines at maturity. Different letters represent significant differences at the 5% level determined by Tukey’s test (*n* ≥ 8). Scale bars: a = 25 cm, b = 3 cm, g = 20 cm, h = 3 cm.

We isolated a natural mutant called *panda* with conspicuous changes in panicle number and grain size. Compared with its wild type parental *japonica* variety ‘Taibei 309’, panicle numbers in the *panda* mutant decreased by ~44.1%, but grain weight increased by ~42.7% in different growth environments (Figure 1; Figures S1 and S2A–C; Table S2). The larger grain size in the mutant *panda* was most likely caused by the increase in cell number rather than cell size in its glume, which is due to a number of the cell cycle‐related genes with significantly higher expression in the 2‐ to 3‐mm young panicles of the *panda* mutant than the wild type (Figure S2D–J). We did not observe consistent changes in spikelet number per panicle and seed‐setting rate in *panda* in multiple environments, indicative of environmental plasticity for these traits (Table S2). These changes in yield‐related traits in the *panda* mutant collectively led to reductions in its yield per plant in most cases (Table S2). In addition, compared with wild type, the *panda* mutant had increased plant height, larger and more drooping leaves, thicker culms, and sometimes longer awns and curly leaves (Figures S1 and S3).

To dissect the genetic basis of the *panda* mutation, we obtained F_1_ plants by crossing *panda* with its wild type parent ‘Taibei 309’, and the F_1_ plants were indistinguishable from wild type in four measured traits (Figure S4). In the F_2_ population obtained by crossing *panda* with the wild type *indica* rice variety ‘Teqing’, the segregation ratio between the wild type and the mutant type with fewer tillers but larger grains was 3:1 (7437:2415, χ^2^ = 0.00042 < χ^2^_0.05,1_ = 3.841, *P* < 0.05). These results indicated that the *panda* mutation was controlled by a single recessive gene.

To finely map the *PANDA* gene, we next conducted genetic mapping using the recombinants from the F_2_ population mentioned above, and finally narrowed the candidate interval down to a 45‐kilobase region, which is flanked by the simple sequence repeat marker (SSR) loci M5 and M7 on chromosome 7 (Figure 1c). We found that there are eight genes within this region, but only the *LOC_Os07g07880* gene had a base change from G to A in the second exon, resulting in an amino acid change from Gly to Asp at the deduced residue 384 (Figure 1d). Thus, we considered this gene to be the candidate gene for *PANDA*.

To confirm the function of the candidate gene, we performed a genetic complementation test by transforming the entire gene including the coding sequence, the 2.6‐kb promoter region and the 1.0‐kb downstream region into the *panda* mutant using agrobacterium‐mediated transformation. All of the independent T_3_‐generation transgenic lines were restored to the wild type based on panicle number and grain size traits (Figure 1a,b,e,f; Figure S5). We also characterized *PANDA*‐overexpressing transgenic plants in which *PANDA* was driven either by the maize *Ubiquitin* promoter in the *japonica* rice cultivar ‘Taipei 309’ background or by its own promoter in the *indica* rice cultivar ‘Genit’ background. Compared with the control, the expression level of *PANDA* in the *Ubiquitin* promoter‐driving transgenic lines increased by ~50 folds, the number of panicles increased by ~35.4%, and the grain weight decreased by ~14.9% (Figure 1g–j). In addition, the transgenic lines decreased in plant height, spikelet per panicle and seed‐setting rate (Figure S6). All these changes collectively resulted in a decrease by 13.9% in grain yield per plant in these transgenic lines (Figures S6 and S8). In the *PANDA* promoter‐driving overexpression lines, the *PANDA* expression level increased by 6.7 folds, panicle number increased by 76.9%, while grain weight decreased by 5.3% (Figures S7 and S8). The moderate increase of *PANDA* expression level in these lines has less negative effects on spikelet number per panicle and seed‐setting per cent, altogether resulting in an increase in yield per plant by 27.7% (Figures S7 and S8). Taken together, these results confirm that *LOC_Os07g07880* is the gene that we named *PANDA*. It is responsible for the balance between panicle number and grain size in rice, and its moderate overexpression has yield increasing potential in rice.

### *PANDA* is expressed broadly and encodes a nuclear‐localized protein

According to the gene expression database CREP (http://crep.ncpgr.cn), the *PANDA* gene is expressed broadly in all rice tissues, such as roots, stems, leaves, young panicles and glumes (Figure S9A). The extensive expression pattern of the *PANDA* gene was verified by qRT‐PCR (Figure S9B). To further confirm this, we transformed the *panda* mutant with a construct consisting of the reporter *GUS* gene fused in frame with the *PANDA* gene driven by of 2.6‐Kb *PANDA* promoter region (*pPANDA::PANDA‐GUS*). The *panda* mutant phenotypes were restored to the wild type in the transgenic plants, suggesting that the fusion protein is functional (Figure S10A–C). We then examined the spatial–temporal glucuronidase (GUS) staining patterns in these plants and observed that the blue GUS stain signal was detectable in most of the rice tissues, such as leaves, glumes and roots (Figure S10D–K).

The PANDA protein is predicted to be a nuclear‐localized protein when analysed with ProtComp 9.0 software (http://linux1.softberry.com). To validate the computational prediction and investigate the subcellular localization of PANDA, we transiently expressed PANDA fused with green fluorescent protein (GFP) (PANDA‐GFP) in rice protoplasts and *N*. *benthamiana* leaves. We observed that the *PANDA‐GFP* fluorescent emissions completely overlapped the NLS‐mCherry signals in the nuclei of rice protoplasts and also in the nuclei of *Nicotiana benthamiana* leaf cells (Figure S11). Taken together, this indicates that the biological function of the *PANDA* protein can be achieved by nuclear localization and being expressed broadly in rice.

### PANDA is a functionally conserved Harbinger transposon‐derived gene

Searching against GenBank and the Repbase database (https://www.girinst.org/repbase), the most comprehensive transposon database including all transposon superfamilies reported thus far (Bao *et al*., 2015), we found that PANDA had significant sequence similarity to the PIF/Harbinger transposons. The protein sequence of PANDA showed 36.1%, 31.1% and 23.4% similarity to the PIF/Harbinger transposons, Harbinger‐4_CMy_1p, Harbinger‐2_AMi_1p and Ping, with *E* values of 3.0E‐47, 3.0E‐34 and 4.0E‐9, respectively (Figure S12A,B). The nucleotide sequence of *PANDA* had a degree of similarity with *Harbinger*‐*4*_*CMy*_*1p* or *Harbinger*‐*2*_*AMi*_*1p*, but almost no similarity with *Ping*. For example, the conserved domain‐encoding nucleotide sequence of *PANDA* showed 68.1% identity with the corresponding part of *Harbinger*‐*2*_*AMi*_*1p* (Figure S12C). Based on comparisons of their protein sequences, rice PANDA and its *Arabidopsis* homolog ALP1 (Antagonist of Like Heterochromatin Protein1) showed closely evolutionary relationships to Harbinger‐2_AMi_1p and Harbinger‐4_CMy_1p than the Ping transposon (Figure S12D). It should be noted that *ALP1* was derived from the evolutionary exaptation of *Harbinger* transposon (Velanis *et al*., 2020). Transposons have terminal inverted repeats and flanking target site duplication, such as *Ping* (Figure S12E), which are the typical features of transposons, while *PANDA* lacked these typical features, suggesting that *PANDA* is a gene without capability for movement in the genome. Additionally, *PIF/Harbinger* transposons can be present in high copy numbers in plant genomes (Casola *et al*., 2007; Grzebelus *et al*., 2007; Jiang *et al*., 2003), while there were only two homologs with high similarity to *PANDA*, *Os11g0702700* and *Os01g0838900* (Figure S13). The two homologs were knocked out using CRISPR/Cas9 system, and the resulting mutants did not exhibit any visible altered phenotypes, such as panicle number and grain size (data not shown), suggesting that the two homologs cannot regulate panicle number and grain size like *PANDA*. Given that *PANDA* showed significant sequence similarity to *Harbinger* transposons and the reported domesticated gene (Velanis *et al*., 2020), the results indicate that *PANDA* is a *Harbinger* transposon‐derived gene.

**Figure 2:**
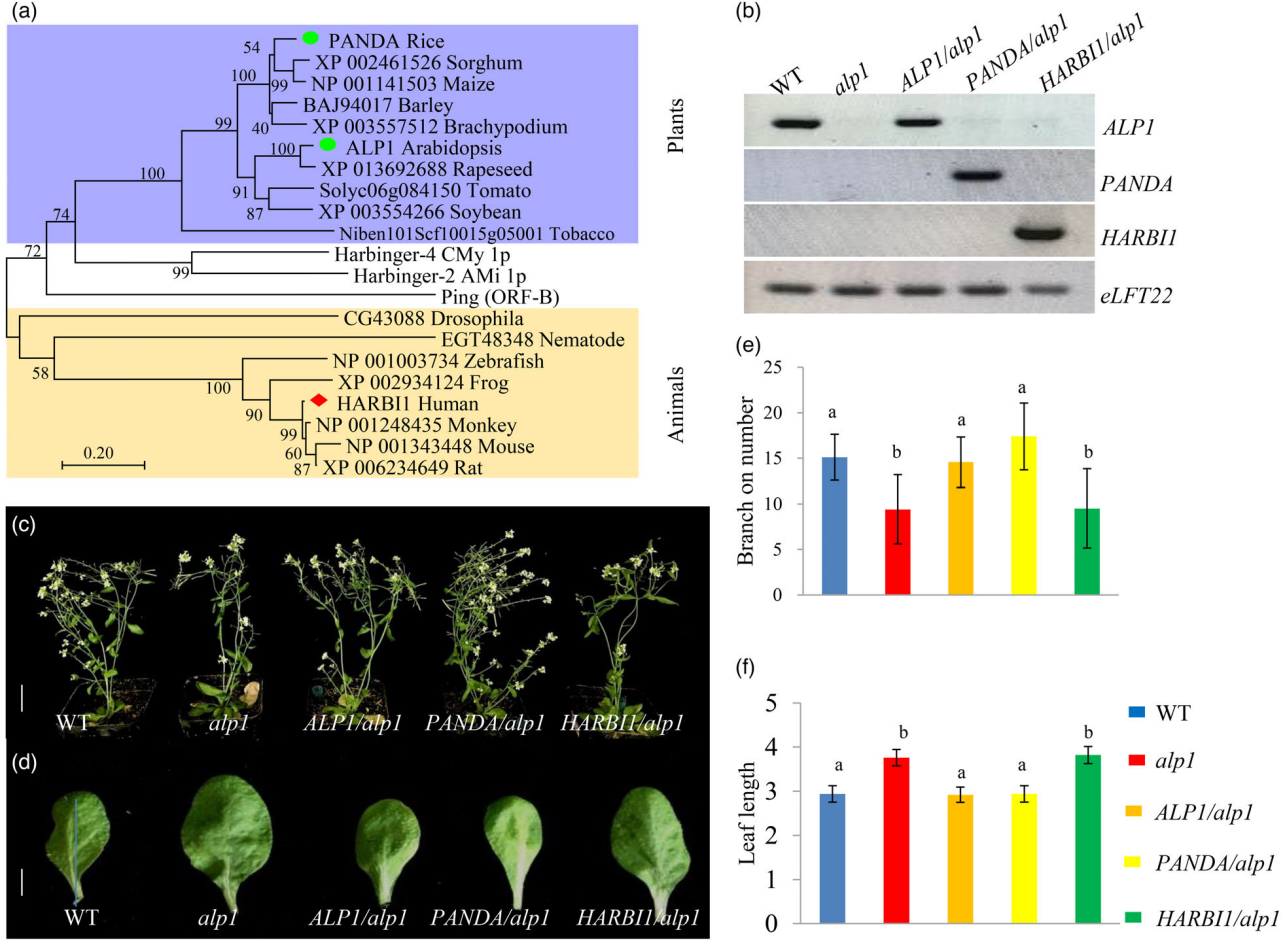
*PANDA* is a functionally conserved *Harbinger* transposon‐derived gene. (a) Phylogenetic tree of PANDA and its homologous proteins in some animals and plants. (b) Semi‐qRT‐PCR analysis of the transgenic lines expressing *PANDA* and two homologous genes in the *Arabidopsis alp1* mutant. (c, d) Branch numbers (c) and leaf size (d) in the transgenic *Arabidopsis alp1* lines expressing *PANDA* homologs. (e, f) Analysis of branch numbers (e) and leaf size (f) in the transgenic lines expressing *PANDA* homologs in the *alp1* mutant background. Scale bars: c = 5 cm, d = 0.75 cm.

The homologs of PANDA were identified in a wide range of organisms including many important crops such as barley (*Hordeum vulgare*), maize (*Zea mays*), soybean (*Glycine max*) and rapeseed (*Brassica napus*) (*E* value <1 × 10^−80^). Impressively, PANDA also showed significant sequence similarity with the proteins in animals including HARBI1, which represents a domesticated PIF/Harbinger transposon in mammals (Kapitonov and Jurka, 2004). To understand the evolutionary relationships between PANDA and other homologous proteins, we conducted a phylogenetic analysis using these homologs and the three PIF/Harbinger transposons mentioned above. PANDA and other plant homologs such as ALP1 in *Arabidopsis* were grouped into a clade, while HARBI1 and other animal homologs were grouped into another clade. Neither clade contained the three transposons (Figure 2a). The wide distributions of the PANDA homologs in plants and their distant phylogenetic relationship with their animal homologs and the three transposons indicated that *PANDA* may be a conserved transposon‐derived gene in rice with neofunctionalization.

Since *PANDA* and its homologs distributed widely in plants, we performed genetic analyses to determine whether they are functionally conserved in plants. We first phenotyped the *Arabidopsis* homolog mutant *alp1*, which has not been well characterized before. Similar to *panda* mutant plants, *alp1* plants showed increases in the sizes of the leaves, flowers and seeds, but had fewer branch numbers compared with the wild‐type *Ler* (Figure 2b–f; Figure S14 and S15). We transformed the rice *PANDA* gene, the *Arabidopsis ALP1* gene and the control animal *HARBI1* gene individually into the *Arabidopsis* mutant *alp1* (Figure 2b). After phenotyping the transgenic lines that showed stable expression, we observed that both the *ALP1*/*alp1* and *PANDA*/*alp1* transgenic lines restored the wild type phenotypes, while the *HARBI1*/*alp1* transgenic lines did not (Figure 2c–f; Figures S14, S15 and S18). Therefore, *PANDA* and *ALP1* are neofunctionalized and functionally conserved Harbinger transposon‐derived genes in plants, which can balance the branch number and organ size in transgenic *Arabidopsis*. However, *PANDA* and *HARBI1* showed divergent functions.

The PANDA protein consists of 441 amino acids (UniPro No.: Q8H572), while *Arabidopsis* ALP1 is 397 amino acids in length (UniProtKB No.: Q94K49‐1). Rice PANDA has an additional 44 amino acid sequence at the N‐terminal end compared with APL1 (Figure S16). In order to determine whether the additional 44 amino acids affect the function of PANDA, we performed genetic complementation by transforming the entire 1,326 base pair *PANDA* ORF or a truncated *PANDA* ORF with a length of 1,194 base pairs fused with the 35S promoter into the rice *panda* and *Arabidopsis alp1* mutants using *Agrobacterium‐*mediated transformation. The T_3_‐generation transformed lines for each transformation combination were characterized, such as *PANDA^441aa^*/*panda*, *PANDA^397aa^*/*panda*, *PANDA^441aa^*/*alp1* and *PANDA^397aa^*/*alp1*. In rice, the transgenic *PANDA^441aa^*/*panda* lines were completely restored to the wild type phenotype, while *PANDA^397aa^*/*panda* plants were showed only partial recovery of the wild type phenotypes, such as complete recovery on panicle number, but partial recovery on grain size and plant height (Figure S17). In *Arabidopsis*, both *PANDA^441aa^*/*alp1* and *PANDA^397aa^*/*alp1* can restore the *alp1* mutant to wild type in terms of branch number and organ size (Figure 2c–f; Figures S14, S15 and S18). Taken together, these results showed that the additional N‐terminal 44 amino acid sequence in the PANDA protein is necessary for the biological function of *PANDA* in rice but not in *Arabidopsis*, suggesting that the sequence divergence between *PANDA* and *ALP1* is likely arisen after the evolutionary split from the common ancestor.

### PANDA interacts with polycomb repressive complex 2 (PRC2) and regulates H3K27me3 deposition in the rice genome

**Figure 3:**
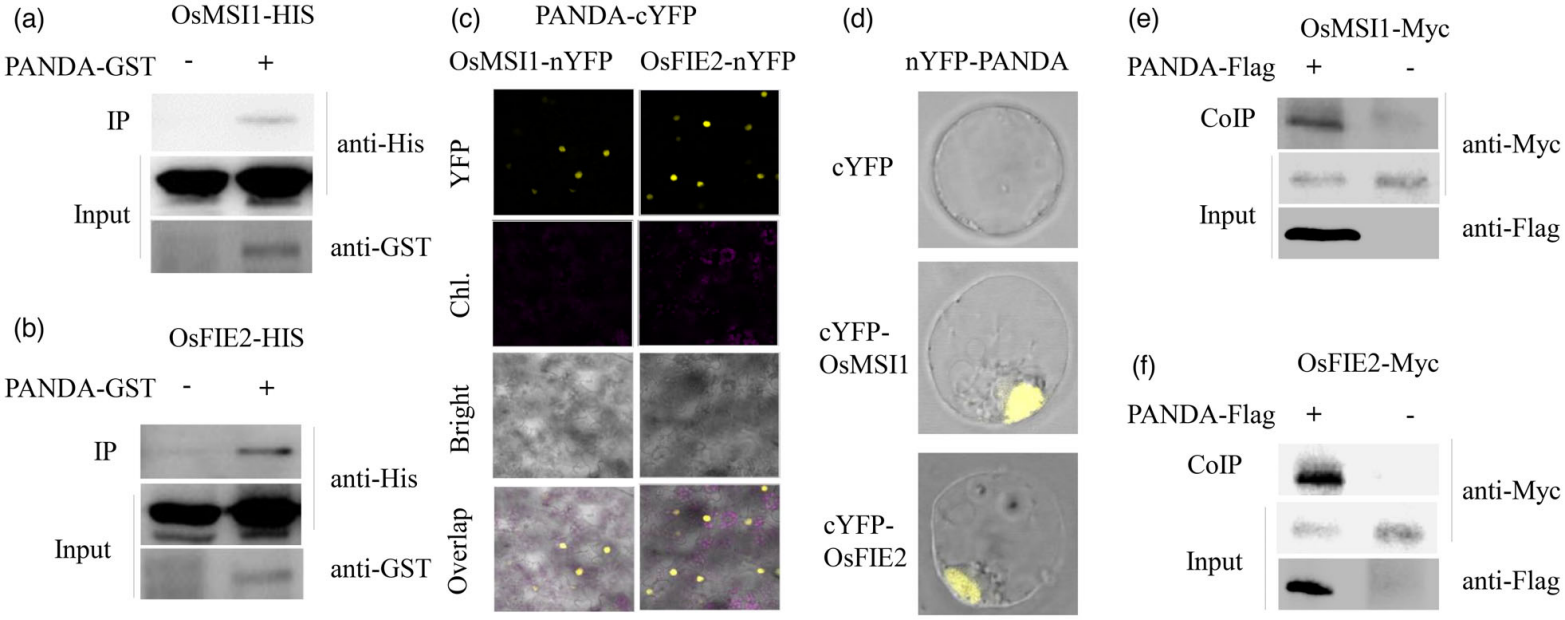
Protein–protein interactions between PANDA and PRC2 components. (a, b) Pull‐down analyses between PANDA and two components of PRC2, OsMSI1 and OsFIE2. (c, d) BiFC analyses between PANDA and OsMSI1 or OsFIE2 in leaf cells of *Nicotiana benthamiana* (c) or rice protoplasts (d). (e, f) CoIP analyses between PANDA and OsMSI1 (e) and PANDA and OsFIE2 (f) in rice protoplasts.

ALP1, a homologous protein of PANDA, in *Arabidopsis*, has been reported to be an accessory protein associated with the MSI1 of PRC2 that is responsible for H3K27me3 formation (Liang *et al*., 2015). This prompted us to assess whether the PANDA protein interacts with the components of PRC2 in rice. An *in vitro* pull‐down assay was performed between PANDA and each of the components of PRC2. As shown in Figure 3a,b, The PANDA‐GST fusion protein can be immunoprecipitated with OsMSI1‐His or OsFIE2‐His *in vitro,* respectively. Bimolecular fluorescence complementation (BiFC) assays and the CoIP test were then performed to confirm the interaction *in vivo*. As shown in Figure 3c,d, a strong fluorescence signal was observed in the nucleus in both rice protoplasts and *N*. *benthamiana* leaves, which co‐expressed the C‐terminal half of YFP fused to the PANDA protein (PANDA‐cYFP) and the N‐terminal half of YFP fused to OsMSI1 (OsMSI1‐nYFP) or OsFIE2 (OsFIE2‐nYFP). The PANDA protein fused to the Flag tag co‐precipitated with the OsMSI1 or OsFIE2 protein with the MYC tag in rice protoplasts (Figure 3e,f). Taken together, these results simply that PANDA interacts with the PRC2 in rice by binding to both OsMSI1 and OsFIE2, the components of the PRC2. Additionally, we also assessed whether the mutation (G384D) of PANDA affects its interaction with the components of PRC2. The results of pull‐down assay showed that both the wild‐type and the mutant PANDA can bind to OsMSI1 and OsFIE2 (Figure S19A,B), suggesting that the mutation of PANDA with amino acid change from Gly to Asp at the position 384 does not affect its binding ability with PRC2.

**Figure 4:**
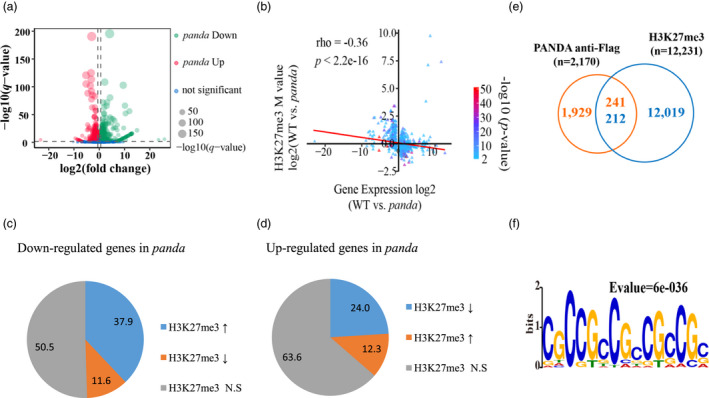
*PANDA* regulates the deposition of H3K27me3 in the rice genome. (a) Differential expression of genes in leaves between wild type and the *panda* mutant. (b) Correlation analysis between RNA levels and H3K27me3 enrichment levels. (c) Percentages of the different H3K27me3 enrichment types of the down‐regulated genes in *panda* (up arrow, higher H3K27me3 in *panda* than wild type; down arrow, lower H3K27me3 in *panda* than wild type; N.S., no significant differences between two genotypes). (d) Percentage of the different H3K27me3 enrichment types of the up‐regulated genes in *panda* (the same as above). (e) Overlapping loci with deposition by H3K27me3 and PANDA‐Flag. (f) The motif enriched in the peaks with deposition of PANDA.

Polycomb repressive complex 2 has been reported to epigenetically repress gene expression through the deposition of H3K27me3 in the rice genome (Bieluszewski *et al*., 2021). To assess the effects of PANDA on gene expression, we firstly carried out RNA‐seq and found that there were 762 up‐regulated genes and 1,236 down‐regulated genes in the *panda* mutant compared with the wild type control (Figure 4a). To assess the repressive role of H3K27me3 in regulating gene expression through PANDA, we also performed anti‐H3K27me3 ChIP‐seq between wild type and *panda* (Figure S20). We found that 2,079 genomic loci exhibited significantly reduced H3K27me3 enrichment levels and 576 loci had significantly induced H3K27me3 enrichment levels in *panda* compared with wild type control (Figure S20A,B). To investigate the relationship between H3K27me3 enrichment levels and the expression levels of all genes in WT and *panda*, we plotted normalized read counts of H3K27me3 ChIP‐seq reads across ±1 kb of TSSs and TTSs of genes with different expression levels (high, medium or low FPKM value) and observed an overall negative correlation between H3K27me3 enrichment levels and the expression levels of all expressed genes in WT and *panda* (Figure S20C,D), which is consistent with repressive roles of H3K27me3 in gene expression. We then analysed the correlation between the change of H3K27me3 enrichment levels and expression levels for all genes between WT and *panda*. We found that the changes of H3K27me3 enrichment level did not exhibit a significant correlation with the expression changes of all genes (Figure S20E). A plausible explanation is that change of H3K27me3 enrichment level may affect expression level of a subset of genes. To test this possibility, we further analysed the relationship for both H3K27me3 and genes with significant changes between WT and *panda*. Indeed, we observed a negative correlation between changes of H3K27me3 enrichment level and expression levels in 978 genes with significant changes both in H3K27me3 enrichment and in expression levels (*rho* = −0.36, *P* < 0.01, Figure 4b). Therefore, the results reflected the repressive role of H3K27me3 in regulating gene expression (Liang *et al*., 2015). In detail, among the up‐regulated genes in *panda*, 63.6% of them had no significant differences in H3K27me3 enrichment levels between *panda* and wild type, while 24.0% and 12.3% of them had reduced and increased H3K27me3 enrichment levels in *panda* compared with wild type, respectively. Among the down‐regulated genes in *panda*, 55.5% had no significant differences in H3K27me3 enrichment levels between two genotypes, while 37.9% and 11.6% of them had increased and reduced H3K27me3 enrichment levels in *panda* relative to wild type, respectively (Figure 4c,d). These genes with a negative correlation between the levels of H3K27me3 enrichment and transcription may be the target genes epigenetically regulated by PANDA. Other differently expressed genes with no differences in H3K27me3 enrichment levels between genotypes may be the genes indirectly regulated by PANDA. All the above analyses suggest that PANDA affects the deposition of H3K27me3 in rice genome.

In order to find the direct *PANDA*‐regulating target genes associated with panicle number and grain size, we carried out anti‐Flag ChIP‐seq analysis using the T_1_‐generation transgenic plants overexpressing the *PANDA‐Flag* fusion gene, which restored the wild type phenotype in the *panda* mutant (Figure 1g–j; Figure S6). We detected a total of 2170 sites with PANDA‐Flag enrichment in the rice genome and 241 loci co‐localized with H3K27me3 enrichment (Figure 4e; Tables S5 and S6). Among the 241 loci, we identified 233 genes, including 52 and 33 genes highly expressed in WT and *panda*, respectively, and 148 genes without significant expression changes between WT and *panda* (Table S7). After conducting a motif enrichment assay, we detected a GCC motif that was significantly enriched in the loci bound by PANDA‐FLAG (Figure 4f). After combining anti‐H3K27me3 and anti‐Flag ChIP‐seq with RNA‐seq data, we found that the growth‐ and development‐related genes bound by PANDA, including *OsMADS22*, *OsMADS55* and *OsEMF1*, had lower H3K27me3 enrichment level but higher transcription levels in the *panda* mutant relative to the wild type (Figure S21, Table S3, S5 and S6). Therefore, we speculate that these growth‐ and development‐related genes are directly epigenetically regulated by the binding of PANDA‐PRC2. In addition, there were other growth‐ and development‐related genes without PANDA deposition but with changes in both the levels of H3K27me3 enrichment and transcription (Table S3, S5 and S6). For example, compared with the wild type, the gene *OsERF112* had higher H3K27me3 deposition but a lower transcription level in the *panda* mutant (Figure S26), suggesting that the gene may be indirectly regulated by PANDA.

### PANDA balances panicle number and grain size by epigenetic silencing of the target genes *OsMADS55* and *OsEMF1*

**Figure 5:**
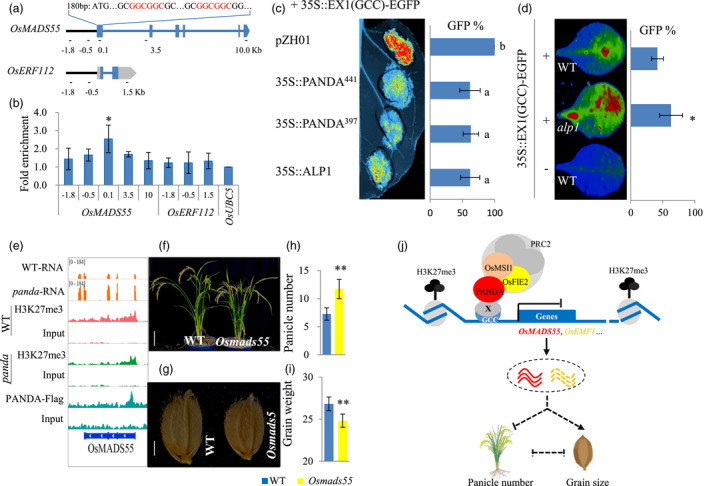
*PANDA* epigenetically regulates the expression of its downstream genes. (a) The distribution of the GCC motif and the fragments of ChIP‐qPCR in the *OsMADS55* or *OsERF112*. (b) ChIP‐qPCR analyses in the *PANDA‐Flag* transgenic liens. (c) PANDA, its truncated form and ALP1 inhibited the expression of GFP fused with the GCC motif‐containing fragment from *OsMADS55* in *N. benthamiana* leaf epidermal cells. (d) The expression of GFP fused with the GCC motif in the *Arabidopsis alp1* mutant and wild type. (e) The combined analysis of the target gene *OsMADS55* by multiple omics (RNA‐seq, anti‐H3K27me3 ChIP‐seq and anti‐PANDA‐Flag ChIP). (F‐I) Panicle numbers (f, h) and grain size (g, i) in the CRISPR/Cas9‐derived mutant *Osmads55* and the wild type. (j) The working model of *PANDA*‐regulating panicle number and grain size. X, a yet‐to‐be‐identified protein or protein complex.

To reveal how PANDA functions in the regulation of rice panicle number and grain size, we specifically analysed the growth‐ and development‐related target genes that were directly epigenetically regulated by PANDA. This group of genes, including *OsMADS22*, *OsMADS55* and *OsEMF1*, exhibited lower H3K27me3 enrichment levels but increased expression levels in the *panda* mutant as compared to wild type (Figures S21 and S22, Table S3). Given that these target genes contain the GCC motif, we inferred that the motif is essential for involvement of PANDA in epigenetically suppressing the expression of the target genes. To prove this hypothesis, we first performed ChIP‐qPCR analysis using the *PANDA‐Flag* transgenic plants. The first exon of *OsMADS55* containing two GCC motifs had significant levels of PANDA‐Flag enrichment, while the *OsERF112* gene without GCC motifs had no significant deposition of PANDA‐Flag (Figure 5a,b). Then, we performed transient expression analyses using reporter genes fused with the first exon of *OsMADS55* containing the GCC motifs *in vivo* (Figure 5c,d). Compared with the negative control, PANDA, its truncated version and its *Arabidopsis* homolog ALP1 significantly inhibited the expression of the fused GFP in leaf cells of *N*. *benthamiana* (Figure 5c). Similarly, we also conducted transient expression of the fused GFP reporter gene in the leaves of wild‐type (*ALP1*) *Arabidopsis* and the *alp1* mutant. We observed that the expression level of GFP in the wild‐type leaves was significantly lower than that in the *alp1* mutant (Figure 5d). When the motifs were mutated, the signals of GFP increased significantly as compared to the wild‐type motif (Figure S23). Therefore, we concluded that the GCC motif is essential for the functions of PANDA in epigenetically silencing its target genes. However, unfortunately, EMSAs failed to support the direct binding of PADNA to the GCC motif (Figure S24). A plausible explanation for this is that PANDA may indirectly bind to the GCC motif, or the epigenetic regulation of PANDA on the GCC motif‐containing target genes may require other protein or protein complex. Additionally, we also assessed the effects of two alleles of PANDA on repressing target gene expression using the GAL4BD system (Lu *et al*., 2013). The wild‐type *PANDA*, the mutant *panda* (G384D), the VP16 positive control and the Flag negative control were fussed to the GAL4 binding domain under the driver of the 35S promoter. Activities of firefly luciferase (LUC) driven by the GAL4 binding element UPSTREAM ACTIVATION SEQUENCE (UAS) were measured using plant in vivo imaging system. As compared to two controls, both GAL4BD‐PANDA and GAL4BD‐panda can inhibit the expression of LUC, but GAL4BD‐panda exhibited less inhibitory effects than GAL4BD‐PANDA (Figure S19C). Considering both genotypes binding to PRC2 as mentioned above, the results suggest that the mutation of PANDA (G384D) likely affects the expression of the target genes through the modified function of the complex.

Through gene editing‐based functional analyses, we found that the *PANDA* target gene, *OsMADS55*, was involved in the regulation of the balance between panicle number and grain size. Both *OsMADS55* and *OsMADS22,* which encode SVP‐group MADS‐box transcription factors, have been reported to act as negative regulators of brassinosteroid responses (Lee *et al*., 2008). Since it has been reported that the knockout of *OsMADS22* did not result in any visible phenotypic changes (Lee *et al*., 2008), we then specifically analysed the CRISPR/Cas9‐mediated knockout of *OsMADS55*. Compared with the wild type control, plants of the *Osmads55* mutant in the *japonica* rice cultivar ‘Zhonghua 11’ had a significant increase (60.3%) in panicle number, a significant decrease (7.5%) in grain weight and also a significant decrease (15.6%) in plant height (Figure 5e–i). The double gene mutations of *OsMADS55* and *PANDA* were obtained by knocking out *OsMADS55* in the *panda* mutant background. Compared with both *panda* and the wild type control, the *pandaOsmads55* double‐mutant plants had more panicles but smaller grains and shorter stems (Figure S25), suggesting that *OsMADS55* functions at the downstream of *PANDA*. In addition, the gene *OsEMF1* bound by PANDA in the *PANDA‐Flag* overexpression plants also had reduced H3K27me3 enrichment level but higher transcription levels in the *panda* mutant relative to wild type (Figures S21 and S22, Table S3). *OsEMF1* encodes a plant‐specific EMF1‐like protein and also has been reported to control rice architecture by regulation of brassinosteroid signalling. The *Osemf1* mutant has been shown previously to have increases in the number of tillers but reduced grain weight (Liu *et al*., 2018; Yan *et al*., 2015; Zheng *et al*., 2015). These genetic analyses further confirmed that *OsMADS55* and *OsEMF1* were involved in the regulation of the balance between panicle number and grain size in rice (Figure 5j).

Additionally, we also genetically analysed the growth and development‐related genes that were indirectly regulated by *PANDA*. We found that *OsERF112* was regulated by *PANDA* and related to grain size. *OsERF112* is predicted to encode an AP2 domain‐containing protein, which functions as an ethylene response factor. This gene had no PANDA deposition in the rice genome, but was more enriched with H3K27me3 and down‐regulated in expression levels in the *panda* mutant relative to wild type (Figure S26A), suggesting that *PANDA* may promote transcription of *OsERF112* by indirectly epigenetic regulation. The *OsERF112* knockout plants did not show any significant change in panicle number, but exhibited a significant increase in grain size (Figure S26B–E), suggesting *OsERF112* only negatively regulates grain size. Therefore, as shown in Figure S26F, *PANDA* promoted the transcription of *OsERF112* indirectly by epigenetic regulation, and then, *OsERF112* negatively regulated the grain size.

Taken together, these results show that PANDA epigenetically coordinates panicle number and grain size by directly silencing its target genes *OsMADS55* and *OsEMF1*, and/or by indirectly regulating the transcription of other downstream genes that are independently responsible for panicle number or grain size.

## Discussion

Both panicle number and grain size are important agronomic traits for rice yield. Considerable progress has been made on understanding the genetic and molecular bases of these two traits independently (Li *et al*., 2018; Wang *et al*., 2018). However, the genetic basis of the balance between panicle number and grain size in rice is still unclear (Fu *et al*., 2010; Guo *et al*., 2020). Dissection of the underlying molecular mechanism will provide a full understanding of the genetic control, which will benefit crop yield improvement. In this study, we characterized an epigenetic regulator *PANDA* balancing panicle number and grain size. The mutation of *PANDA* showed reduced panicle numbers and increased grain size (Figure 1a,b,e,f; Figures S1–S5), while *PANDA* overexpression lines showed an increase in panicle number but a decrease in grain size (Figure 1g–j; Figures S6 and S7). Analyses of genome‐wide histone modification, RNA‐seq and ChIP‐seq found that a set of genes were epigenetically regulated (Figure 4; Figure S20; Tables S3–S6). Knocking out of the growth‐ and development‐related target genes of *PANDA*, such as *OsMADS55* and *OsEMF1*, reversed the mutant *panda* phenotype on panicle number and grain size (Figure 5f–i; Figure S25; Liu *et al*., 2018). *OsMADS55* is a negative BR‐responsive gene (Lee *et al*., 2008). OsEMF1 can interact with OsARF11 and then bind to the *OsBRI1* promoter to modulate BR signalling (Liu *et al*., 2018). The role of BR on the control of grain size has been shown in a number of BR biosynthesis and signalling mutants (Li *et al*., 2018). Recently, it was reported that BR is involved in rice panicle formation by regulating the stability of the D53‐OsBZR1 complex to regulate FC1 expression (Fang *et al*., 2020). Therefore, this suggests that PANDA balances panicle number and grain size through the BR‐dependent pathway. Consistently, an allele of *PANDA*, *POW1*, is involved in separable regulation of grain size and leaf angle development through the BR‐dependent signal pathway in rice (Zhang *et al*., 2021). However, we should note that other genetic factors, such as *OsERF112*, also contribute to the phenotypic changes in grain size independently. Knockout of *OsERF112* partly phenocopied the *panda* mutant on grain size, but not on panicle number (Figure S26). Altogether, *PANDA* epigenetically coordinates panicle number and grain size in a BR‐dependent way, while BR‐independent factors that work downstream of PANDA also contribute to the phenotypic changes separately.

PANDA and its homolog ALP1 in *Arabidopsis* associate with PRC2 to modify the histone methylation and epigenetically repress expression of its target genes (Figures 3, 4, 5; Liang *et al*., 2015; Velanis *et al*., 2020). Our ChIP‐seq analyses showed a GCC motif that was significantly enriched in the regions of PANDA deposition (Figures 4f and 5a–d). However, it is still an open question as to how PANDA modulates the recruitment of the PRC2 complex to target loci containing this motif. A similar motif has been reported to be associated with H3K27me3 deposition in *Arabidopsis* (Xiao *et al*., 2017). This motif in rice can be recognized and bound by the transcription factor NGR5 to recruit PRC2 to modify the histone methylation and repress expression of its target genes (Wu *et al*., 2020). We did not obtain evidence showing a direct interaction of PANDA with NGR5 or its partner LC2 by yeast two‐hybrid assays (data not shown). Although PANDA has an HTH domain, which is a DNA‐recognition motif often involved in DNA binding (Zaveri *et al*., 2021), PANDA did not bind to the GCC motif through the HTH domain directly (Figure S24). The results suggest that the epigenetic regulation of PANDA on the GCC motif‐containing target genes, including *OsMADS55* and *OsEMF1*, may require a yet‐to‐be‐identified protein or protein complex. Histone methylation can be either established by accessary protein‐mediated PRC2 deposition, or maintained and re‐established by PRC1 during chromosome duplication (Blackledge *et al*., 2014). ALP1 antagonizes LHP1, which is incorporated into the PRC1 complex functioning as a histone methylation reader and maintainer (Liang *et al*., 2015; Tao *et al*., 2019; Yuan *et al*., 2016). Both PANDA and LHP1 can bind to MSI1 in rice or in *Arabidopsis* (Figure 3; Derkacheva *et al*., 2013). As several independent PRC1 complexes have been reported to coordinate genome‐wide gene expression (Li *et al*., 2018), it is tempting to assume that PANDA incorporates in a PRC1 complex independent of OsLHP1 to modulate the PRC2 recruitment and gene silencing. Analysis of the PANDA interactome will provide a full picture of its genetic network and facilitate a deep understanding of epigenetic regulation in plants.

Transposons were often considered to be ‘junk DNA’ or ‘genome parasites’, but they can be ‘domesticated’ and evolve new cellular functions that benefit the host (Kapitonov and Jurka, 2004; Smit and Riggs, 1996). Thus far, most domesticated transposons were found in mammals, and only a few cases of transposon domestication have been reported in plants (McDowell and Meyers, 2013; Volff, 2006). Our results indicated *PANDA* showed significant sequence identity to *Harbinger* transposons and reported transposon‐related genes, suggesting that *PANDA* is likely derived from a *Harbinger* transposon but evolved into a new gene due to loss of its mobility and undergoing neofunctionalization that controls panicle number and grain size in rice. Moderate overexpression of *PANDA* under its own promoter in the *indica* background can significantly increase grain yield per plant (Figures S7 and S8). Therefore, *PANDA* is a potential gene for rice yield improvement in future. Homologs of *PANDA* were also present in many other important crops (Figure 2a) and showed high sequence similarity to *PANDA*. It is worthwhile to further address whether the homologs of *PANDA* in these crops also govern phenotypic traits similar to panicle number and grain size. Since the homologs of *PANDA* were found in diverse plants including both dicots and monocots, which split from a common ancestor over 150 million years ago (MYA) (Zeng *et al*., 2014), and *PANDA* can restore the wild type phenotype in the *Arabidopsis alp1* mutant (Figure 2), this suggests that *APL1* in *Arabidopsis* and *PANDA* in rice may be derived from the same ancient ancestor >150 MYA. The human HARBI1 protein cannot restore the wild type phenotype in *Arabidopsis alp1* and was grouped into a different clade from APL1/PANDA (Figure 2b). It is possible that the HARBI1 and other homologous proteins in animals were domesticated independently. However, more analyses are necessary to further address the evolution and functional divergence of *PANDA*‐related genes in plants.

In conclusion, we have identified a transposon‐derived gene with neofunctionalization that epigenetically coordinates panicle number and grain size in rice. Our results also provide novel insights into the genetic control of rice yield traits and a potential way to improve crop yields associated with this gene.

## Materials and methods

### Plant materials and field experiments

The rice mutant ‘*panda*’ was a natural variant of the *japonica* rice variety ‘Taibei 309’. The F_2_ population used for map‐based cloning was obtained by crossing the mutant ‘*panda*’ with the *indica* rice variety ‘Teqing’, followed by self‐pollination of the F_1_ hybrid. The *japonica* rice cultivars ‘Taibei 309’ (TB309) and ‘Zhonghua 11’, and the *indica* rice cultivar ‘Genit’ were used for genetic transformation. Plants were cultivated in paddy fields following normal agricultural practices with a row spacing of 20 cm, a plant spacing of 15 cm, and 10 plants per row at our experimental farms in Sanya, Changsha and Beijing during the winter or the summer of 2014–2020. More than 10 plants of each line were used to dissect the yield‐related traits. The significant differences were determined by Student’s *t*‐test or Tukey’s honestly significant difference (HSD) test.

To test the functional conservation of *PANDA* in plants, we collected the mutant *alp1* and its wild type (Ler) of *Arabidopsis thaliana*. The *Arabidopsis* plants were cultivated in a growth chamber (Percival Scientific, Perry, IA) under a 16‐h day/8‐h night photoperiod at temperatures of 23 °C (day) and 20 °C (night).

### Map‐based cloning

In the F_2_ mapping population, we constructed two DNA pools composed of 30 wild‐type plants or 30 mutant‐type plants. The pools were genotyped using genome‐wide SSR markers for the primary mapping of *PANDA*. To fine‐map *PANDA*, 2,415 individual plants with the mutant phenotype were genotyped using the newly developed markers around the primary *PANDA* mapping interval. All genes in the fine‐mapped region were amplified by PCR, and the resulting fragments were subjected to Sanger DNA sequencing. The sequences from both wild type and the *panda* mutant were then compared using the Sequencher 5.0 software (Gene Codes Corporation, Ann Arbor, Michigan) to investigate sequence divergence and identify the candidate gene of *PANDA*.

### Protein sequence analyses

The functional domains present in the PANDA protein were predicted using the Conserved Domain Database (https://www.ncbi.nlm.nih.gov/cdd). The proteins homologous to PANDA were identified using BLASTP searches against the NCBI database (https://www.ncbi.nlm.nih.gov) and the rice genome annotation database (http://rice.uga.edu). An alignment of the homologous protein sequences was performed using the software Clustal W, and a neighbour‐joining phylogenetic tree was constructed using MEGA 5.0 (https://www.megasoftware.net).

### Plasmid construction and plant transformation

Gene fragments were amplified from genomic DNA of ‘Taibei 309’ and then cloned into the binary vectors based on homologous recombination technology using the ClonExpress Entry One Step Cloning Kit (Vazyme, Nanjing, China). The recipient vectors were pTCK303 for constitutive overexpression of *PANDA* driven by the maize *Ubiquitin* promoter (*pUBI*:*PANDA*), pCAMBIA1300 for the complementation assay in the *panda* mutant or moderate overexpression in the *indica* rice cultivar ‘Genit’ driven by its own promoter (*pPANDA*::*PANDA*), and pCAMBIA1301 for the analysis of the *PANDA* promoter activity (*pPANDA*::*PANDA‐GUS*). To obtain the CRISPR/Cas9 knockout lines, the Cas9 gene was driven by the CaMV 35S promoter, while the 20 bases upstream of the protospacer adjacent motifs (PAMs) were selected as target sequences, and their expression was driven by the *OsU3* promoter in the binary vector of BGK03. The target regions in the genome of T_0_‐ and/or T_1_‐generation transgenic plants were sequenced using the related PCR products. All plasmid vectors were introduced into *Agrobacterium tumefaciens* strain EHA105 followed by *Agrobacterium*‐mediated transformation of *japonica* rice cultivars ‘Taibei 309’, ‘Zhonghua 11’, the mutant ‘*panda*’ or the *indica* rice cultivar ‘Genit’. For complementation assays in the *Arabidopsis* mutant *alp1*, the full‐length coding regions of *ALP1*, *PANDA* or *HARBI1* were cloned into the plant binary vector pZH01 to generate *35S*::*PANDA*, which was then used to transform the *Arabidopsis* mutant *alp1* by *Agrobacterium*‐mediated transformation.

### Subcellular localization

The vectors *35S*::*PANDA‐EGFP* and *35S*::*EGFP*, and the nuclear localization marker 35S::*NLS‐mCherry* were introduced into rice protoplast cells by PEG‐mediated transformation or into leaves of *N*. *benthamiana* by *Agrobacterium*‐mediated transformation. Green and red fluorescence signals were observed with a laser confocal microscope (LSM880, Zeiss Corporation, Oberkochen, Germany).

### β‐Glucuronidase staining

Different tissues of the *pPANDA::GUS* transgenic plants were used for β‐GUS staining assays. The reaction solution was 100 mm sodium phosphate, 10 mm EDTA, 0.1% Triton X‐100 and 1 mm 5‐bromo‐4‐chloro‐3‐indolyl‐β‐glucuronic acid (Sigma‐Aldrich, St. Louis, Missouri), pH 7.0. Tissues were stained overnight at 37 °C and then cleared in 75% ethanol. The GUS‐stained signals were finally observed and photographed using a fluorescence microscope (BX51, Olympus Corporation, Tokyo, Japan).

### ChIP assays

The anti‐H3K27me3 ChIP‐seq assay was performed using the *panda* mutant and its wild type cultivar ‘Taibei 309’. The PANDA ChIP‐seq assay was performed using plants expressing *pUBI*::*PANDA‐Flag* in the *japonica* rice cultivar ‘Taibei 309’. Samples of leaf tissue (~4 g) or ~1.0‐cm young panicles were cross‐linked in 1% formaldehyde under vacuum, and the cross‐linking was stopped by adding glycine to a final concentration of 0.125 m. The samples were ground to fine powders in liquid nitrogen for nuclei preparation. After chromatin preparation according to the method described previously (Zhao *et al*., 2020), anti‐H3K27me3 or anti‐Flag polyclonal antibody (#A16199, ABclonal, Wuhan, China; #14793, Cell Signaling Technology, Danvers, Massachusetts) was used to immunoprecipitate the corresponding protein–DNA complexes, and the precipitated DNA was recovered for library preparation followed by sequencing on the Illumina platform. ChIP‐seq data analysis was performed as previously described (Lu *et al*., 2013). The H3K27me3 or PANDA‐Flag ChIP‐qPCR assays were performed using plants expressing *pUBI::PANDA‐Flag* and the wild‐type *japonica* rice cultivar ‘Taibei 309’. Chromatin precipitated with normal mouse IgG was used as the negative control. The precipitated DNA was recovered and analysed by ChIP–qPCR with the primers listed in Table S1.

### RNA sequencing and data analyses

Whole‐transcriptome comparison analyses between the *panda* mutant and the wild type were performed using the leaves of 3‐week‐old plants or about 1‐cm young panicles. Total RNA was extracted with TRIzol reagent (Sangon Biotech, Shanghai, China). The cDNA libraries were constructed following Illumina standard protocols and sequenced on an Illumina HiSeq instrument by Novogene Biotech Co. Ltd. in Beijing city of China. RNA‐seq reads were aligned to the rice reference genome (http://rice.uga.edu) using TopHat after filtering out low‐quality reads (lowest base score <20) using SeqPrep and Sickle (Trapnell *et al*., 2009). Gene expression levels were calculated and normalized to FPKM (fragments per kilobase of transcript per million mapped reads) with HTSeq (Anders *et al*., 2015). Differential gene expression levels were examined using the R package DEGSeq (Wang *et al*., 2010). The cut‐off for significant differential expression was set as log2 (fold‐change) ≥1 and FDR < 0.05.

### qRT‐PCR

Total RNA was extracted from plant tissues using TRIzol reagent (Sangon, Shanghai, China), and the mRNA was reverse‐transcribed into cDNA using the M‐MLV reverse transcriptase kit (Vazyme, Nanjing, China) following the manufacturers’ instructions. Quantitative RT‐PCR (qRT‐PCR) was performed using SYBR I Premix ExTaq (Vazyme, Nanjing, China). The gene expression levels in at least three biological replicates were calculated using the ∆∆*C*_t_ method. Student’s *t*‐test was used to determine significant differences between samples.

### Pull‐down assay

The *PANDA* CDS and the CDS of its interacting protein gene were cloned into the vectors pGSTA or pHISK for fusion with the GST or His tag, respectively. The GST or His fusion proteins were expressed in *Escherichia coli* BL21 and purified using Glutathione Sepharose 4 FF (#175132‐01 GE Healthcare, Chicago, Illinois) and NI‐NTA SefinoseTM Resin (C600033‐0010 BBI South Wales, UK) kits. Pull‐down assays were performed with the Pierce™ GST Protein Interaction Pull‐Down Kit (#21516 Thermo Scientifc, Waltham, Massachusetts).

### BiFC assay

For the BiFC assay, the CDS of *PANDA* or its interacting protein genes was amplified and cloned into the binary vectors pC1300S‐nYFP and pC2300‐cYFP for fusion with nYFP and cYFP, respectively. These vectors were then co‐transformed into rice protoplasts or into *N*. *benthamiana* leaves using the PEG or agrobacterium‐mediated methods for transient expression. The transformed cells were finally observed with a laser confocal microscope (LSM880, Zeiss Corporation, Oberkochen, Germany).

### CoIP assay

The CDS of *PANDA* or its interacting proteins was cloned into the pC1300S‐Flag and pC2300S‐Myc vectors to produce Flag and Myc fusion protein. The vectors were co‐transformed into rice protoplasts for transient expression of these fusion proteins. For the CoIP assay, total protein was extracted from the rice protoplasts. Following the manufacturer’s instructions, 30 μL of the agarose‐conjugated anti‐Flag monoclonal antibody (Sangon, Shanghai, China) was added to 500 μL total extracted proteins and incubated at 4 °C for 3 h with gentle rotation. The beads were washed three times with 350 μL of extraction buffer, and the proteins were eluted with 30 μL SDS‐PAGE sample buffer. Immunoblotting was then performed as previously described (Lu *et al*., 2013). The antibodies we used are as follows: anti‐flag rabbit antibody (#D110005‐0100, Sangon, Shanghai, China), anti‐Myc rabbit antibody (#D110006‐0100, Sangon, Shanghai, China) and HRP‐conjugated goat anti‐rabbit IgG (#D110053‐0025, Sangon, Shanghai, China).

## Conflict of interest

The authors declare no competing interests.

## Author contributions

D.M., D.Z., W.Z. and C.C. designed the research; D.M., S.T., X.L., M.T., C.L., D.W., L.B., Z.H., X.W., L.Y., Y.Z. and D.Z. performed experiments; D.M., D.G. and S.T. analysed the data; and D.M., D.G., W.Z. and C.C. wrote the manuscript.

## Supporting information


**Figure S1** Phenotypic characteristics of the rice *panda* mutant.
**Figure S2** Statistical analyses of the grain size traits and the related gene expression levels between wild type (WT) and the mutant *panda*.
**Figure S3** Statistical analyses of leaf length, leaf width, and leaf angle between wild type (WT) and *panda*.
**Figure S4** Statistical analysis of plant height, panicle number, grain number, seed setting rate, grain weight, and grain yield among the wild type *japonica* cultivar ‘TB309’, the mutant *panda*, and their hybrid F_1_.
**Figure S5** Statistical analyses of panicle number, grain number, seed setting rate, grain weight, and grain yield between the wild type cultivar ‘TB309’, the mutant *panda*, and the transgenic *pPANDA*::*PANDA*/*panda* complementation lines in the *panda* genetic background.
**Figure S6** Expression and phonotype analyses of the constitutive *PANDA‐*overexpression lines.
**Figure S7** Expression and phonotype analyses of the moderate *PANDA‐*overexpression lines.
**Figure S8** Comparison of changes in expression and yield‐related traits between two kinds of the *PANDA* overexpression lines.
**Figure S9** Expression pattern of *PANDA* in rice tissues at various developmental stages.
**Figure S10** GUS staining of *pPANDA::PANDA‐GUS* transgenic plants.
**Figure S11** Subcellular localization of PANDA.
**Figure S12** Homology analyses between PANDA and the PIF/Harbinger transposons.
**Figure S13** Phylogenetic tree of PANDA homologous proteins in rice.
**Figure S14** Genetic complementation in the *Arabidopsis alp1* mutant by using the rice *PANDA* gene.
**Figure S15** Seed sizes in wild‐type *Arabidopsis*, the *alp1* mutant, and the *35S::PANDA* transgenic line in the mutant *alp1* background.
**Figure S16** Protein structures of PANDA and ALP1.
**Figure S17** The N‐terminal 44 amino acids are necessary for PANDA to completely rescue the mutant phenotype in rice.
**Figure S18** Comparisons of the phenotypes of the whole plant (A), flowers (B), and leaves (C) in WT, *alp1*, and two transgenic lines overexpressing the truncated 397 aa‐length PANDA; *PANDA^397^*/*alp1* #1 and *PANDA^397^*/*alp1* #2. Scale bars in A = 3.5 cm, B = 4 cm, and C = 0.5 cm.
**Figure S19** Function diversity between wild type and the mutant of *PANDA*.
**Figure S20** anti‐H3K27me3 ChIP‐seq analysis between wild type and *panda*.
**Figure S21** RNA‐seq, anti‐H3K27me3 ChIP‐seq, and anti‐Flag ChIP‐seq analyse of target genes of *PANDA*, such as *OsMADS22*, *OsMADS55* and *OsEMF1*.
**Figure S22** qRT‐PCR analyses of *PANDA*, *OsMADS55*, *OsEMF1, OsMADS22* and *OsERF112* in wild type (WT) and the *panda* mutant.
**Figure S23** PANDA inhibit the expression of GFP fused with the first exon of *OsMADS55* containing GCC motifs in *N. benthamiana*.
**Figure S24** Electrophoretic Mobility Shift Assay (EMSA) of PANDA binding to the GCC motif.
**Figure S25** Analyses of plants carrying the double mutation in *PANDA* and its target gene *OsMADS55*.
**Figure S26** Genetic analyses of the relationship between *PANDA* and *OsREF112*.

**Table S1** Primers used in this study.
**Table S2** Phenotypic analysis of the rice *panda* mutant and the wild type in the different environments.
**Table S3** List of all genes with changes in the level of RNA or H3K27me3 enrichment.
**Table S4** List of the groups with different H3K27me3 enrichment levels in the genes regulated by PANDA.
**Table S5** List of all genes with deposition of PANDA‐Flag in the first ChIP‐seq.
**Table S6** List of all genes with deposition of PANDA‐Flag in the second ChIP‐seq.
**Table S7** List of the 241 PANDA‐FLAG binding sites co‐localized with H3K27me3 enrichment sites.
