# Supplementary material for: The Harbinger transposon‐derived gene PANDA epigenetically coordinates panicle number and grain size in rice
Source: Plant Biotechnol J. 2022 Mar 16;20(6):1154–66. doi: 10.1111/pbi.13799 (PMC9129072; doi:10.1111/pbi.13799)
Supplement: Supplementary file 1 — Figure S1 Phenotypic characteristics of the rice panda mutant. Figure S2 Statistical analyses of the grain size traits and the related gene expression levels between wild type (WT) and the mutant panda. Figure S3 Statistical analyses of leaf length, leaf width, and leaf angle between wild type (WT) and panda. Figure S4 Statistical analysis of plant height, panicle number, grain number, seed setting rate, grain weight, and grain yield among the wild type japonica cultivar ‘TB309’, the mutant panda, and their hybrid F1. Figure S5 Statistical analyses of panicle number, grain number, seed setting rate, grain weight, and grain yield between the wild type cultivar ‘TB309’, the mutant panda, and the transgenic pPANDA::PANDA/panda complementation lines in the panda genetic background. Figure S6 Expression and phonotype analyses of the constitutive PANDA‐overexpression lines. Figure S7 Expression and phonotype analyses of the moderate PANDA‐overexpression lines. Figure S8 Comparison of changes in expression and yield‐related traits between two kinds of the PANDA overexpression lines. Figure S9 Expression pattern of PANDA in rice tissues at various developmental stages. Figure S10 GUS staining of pPANDA::PANDA‐GUS transgenic plants. Figure S11 Subcellular localization of PANDA. Figure S12 Homology analyses between PANDA and the PIF/Harbinger transposons. Figure S13 Phylogenetic tree of PANDA homologous proteins in rice. Figure S14 Genetic complementation in the Arabidopsis alp1 mutant by using the rice PANDA gene. Figure S15 Seed sizes in wild‐type Arabidopsis, the alp1 mutant, and the 35S::PANDA transgenic line in the mutant alp1 background. Figure S16 Protein structures of PANDA and ALP1. Figure S17 The N‐terminal 44 amino acids are necessary for PANDA to completely rescue the mutant phenotype in rice. Figure S18 Comparisons of the phenotypes of the whole plant (A), flowers (B), and leaves (C) in WT, alp1, and two transgenic lines overexpressing the truncated 397 aa‐length [file PBI-20-1154-s001.pdf]

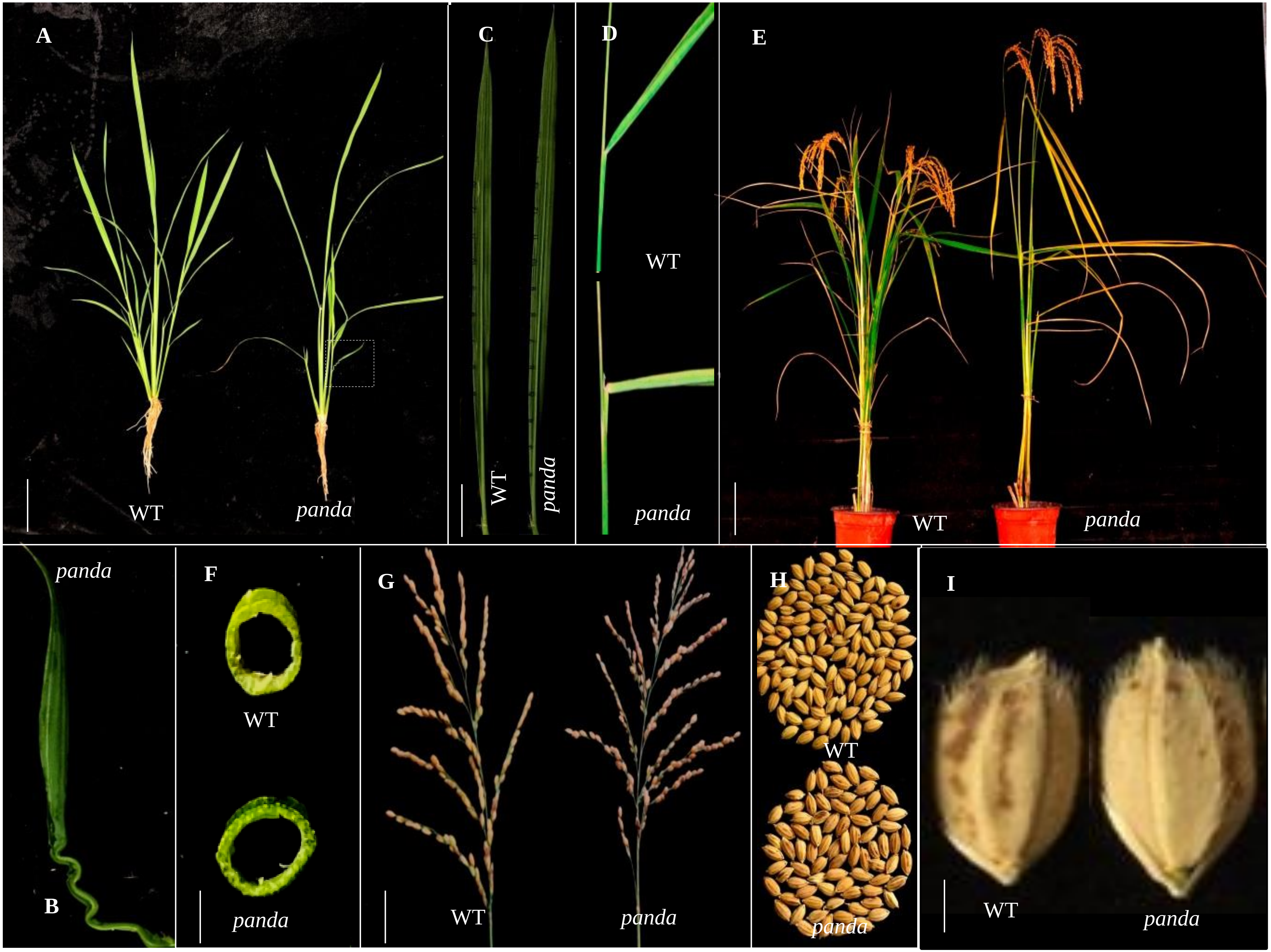
A
WT
panda
C
WT
panda
D
WT
panda
E
WT
panda
panda
B
F
WT
panda
G
WT
panda
H
WT
panda
I
WT
panda

Fig. S1

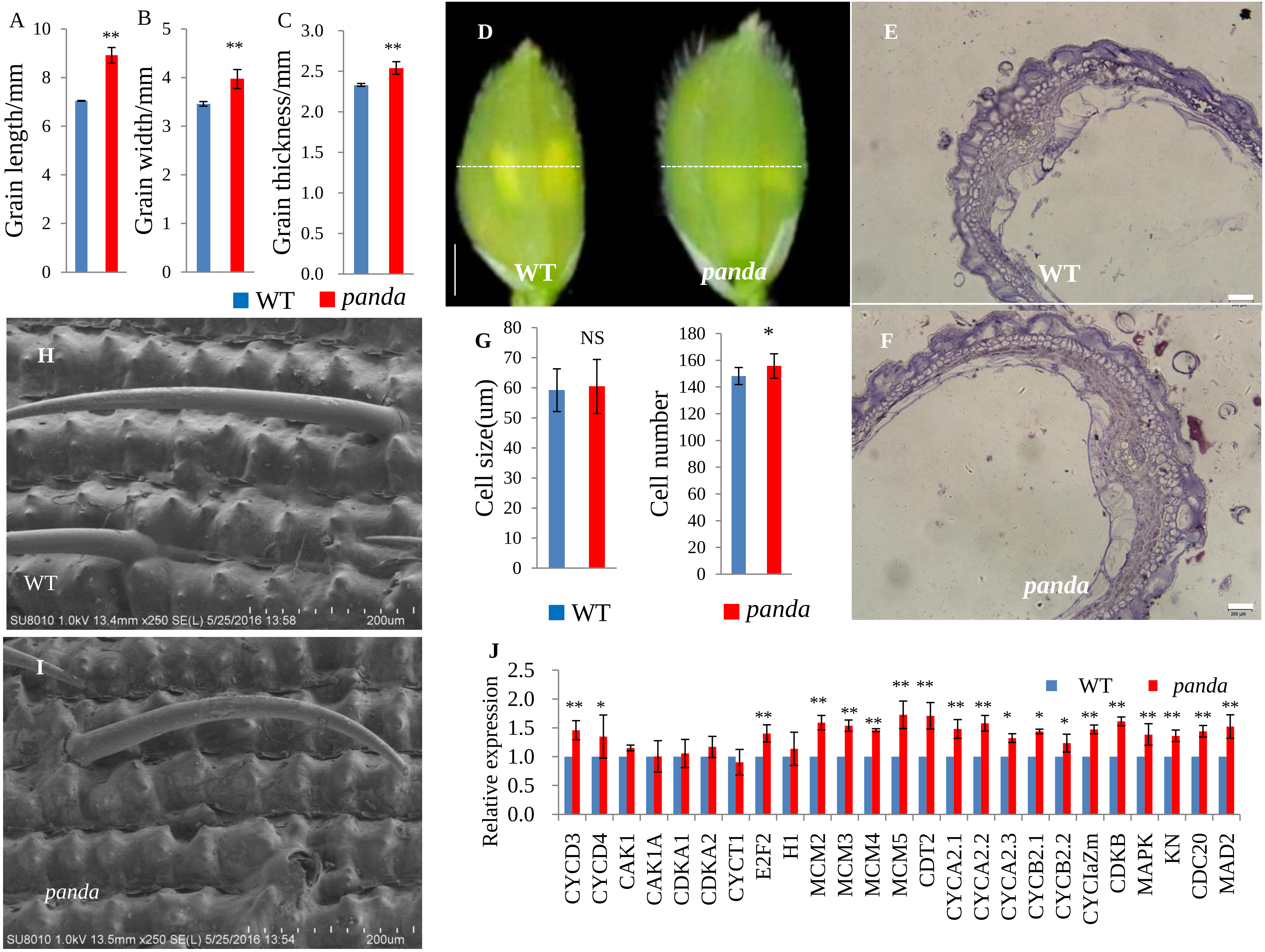
A
Grain length/mm
**
B
Grain width/mm
**
C
Grain thickness/mm
**
WT
panda
D
WT
panda
E
WT
F
panda
G
Cell size(um)
NS
Cell number
*
WT
panda
H
WT
SU8010 1.0kV 13.4mm x250 SE(L) 5/25/2016 13:58
200um
I
panda
SU8010 1.0kV 13.5mm x250 SE(L) 5/25/2016 13:54
200um
J
Relative expression
WT
panda
CYCD3
CYCD4
CAK1
CAK1A
CDKA1
CDKA2
CYCT1
E2F2
H1
MCM2
MCM3
MCM4
MCM5
CDT2
CYCA2.1
CYCA2.2
CYCA2.3
CYCB2.1
CYCB2.2
CYCIaZm
CDKB
MAPK
KN
CDC20
MAD2

Fig. S2

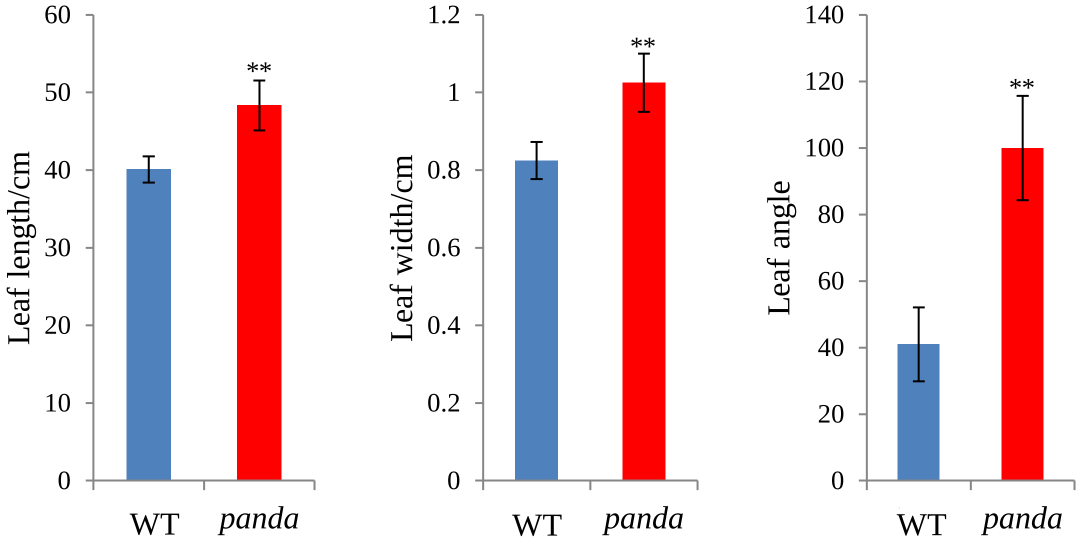

60
50
40
30
20
10
0
Leaf length/cm
**
WT
panda
1.2
1
0.8
0.6
0.4
0.2
0
Leaf width/cm
**
WT
panda
140
120
100
80
60
40
20
0
Leaf angle
**
WT
panda

Fig. S3

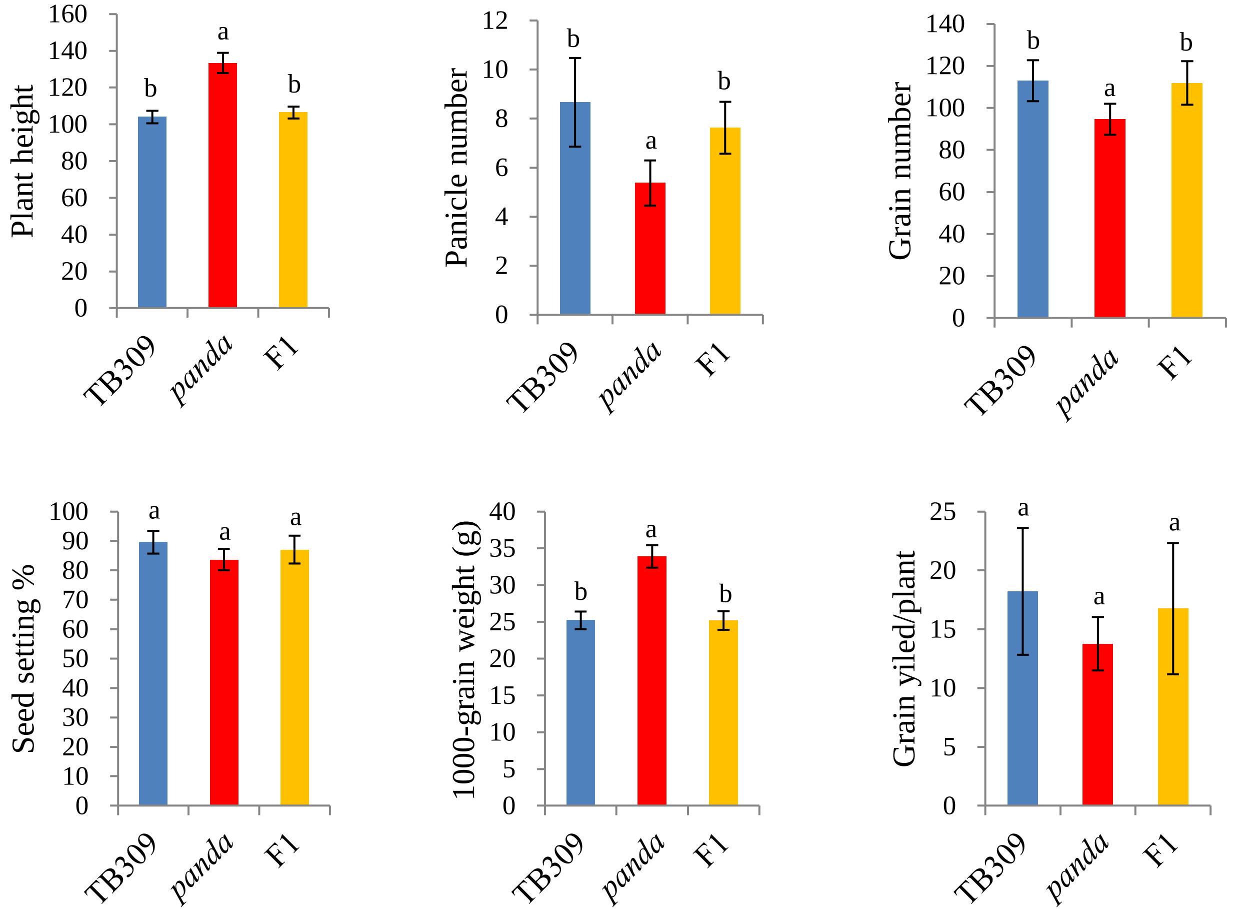
Plant height
160
140
120
100
80
60
40
20
0
b
a
b
TB309
panda
F1
Panicle number
12
10
8
6
4
2
0
b
a
b
TB309
panda
F1
Grain number
140
120
100
80
60
40
20
0
b
a
b
TB309
panda
F1
Seed setting %
100
90
80
70
60
50
40
30
20
10
0
a
a
a
TB309
panda
F1
1000-grain weight (g)
40
35
30
25
20
15
10
5
0
b
a
b
TB309
panda
F1
Grain yiled/plant
25
20
15
10
5
0
a
a
a
TB309
panda
F1

Fig. S4

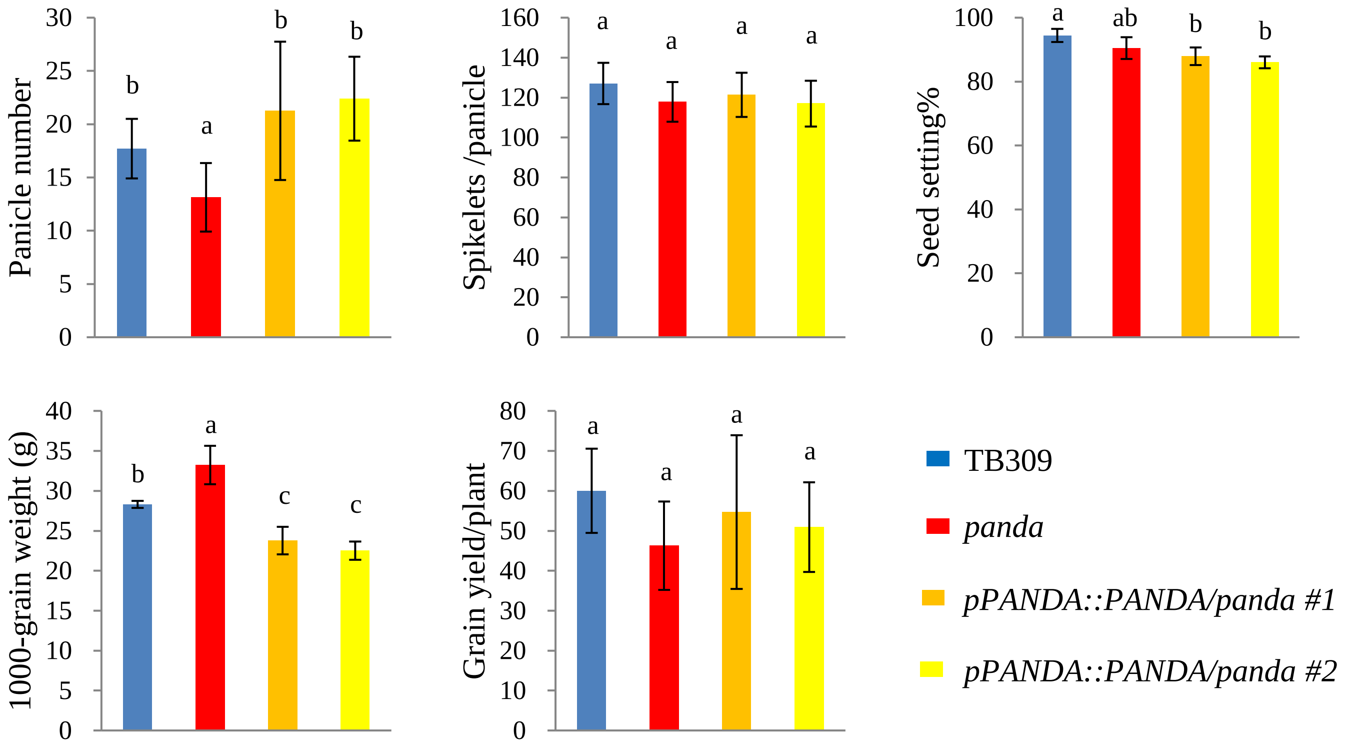
Panicle number
Spikelets /panicle
Seed setting%
1000-grain weight (g)
Grain yield/plant
TB309
panda
pPANDA::PANDA/panda #1
pPANDA::PANDA/panda #2

Fig. S5

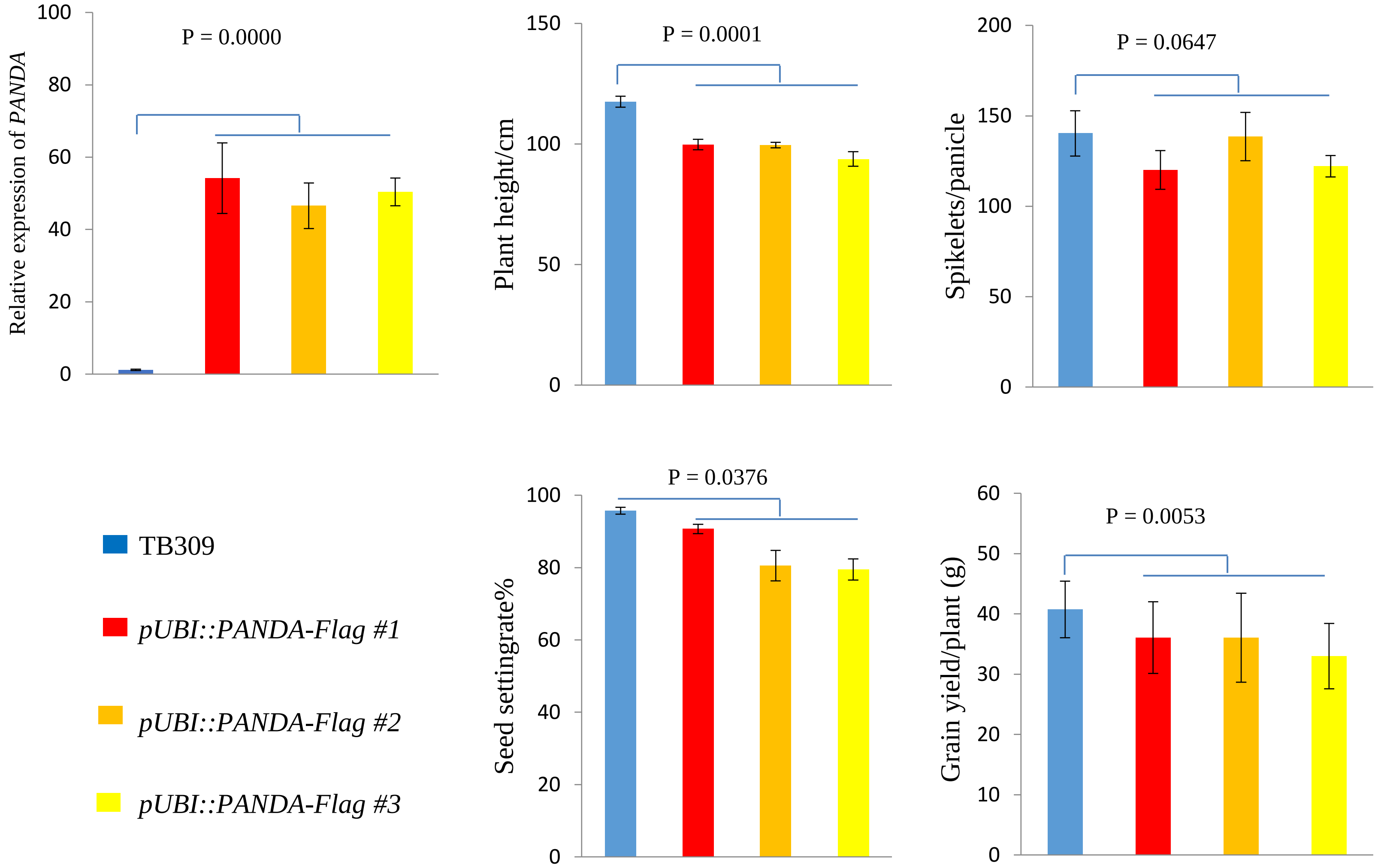
P = 0.0000
Relative expression of PANDA
100
80
60
40
20
0
P = 0.0001
Plant height/cm
150
100
50
0
P = 0.0647
Spikelets/panicle
200
150
100
50
0
P = 0.0376
Seed settingrate%
100
80
60
40
20
0
P = 0.0053
Grain yield/plant (g)
60
50
40
30
20
10
0
TB309
pUBI::PANDA-Flag #1
pUBI::PANDA-Flag #2
pUBI::PANDA-Flag #3

Fig. S6

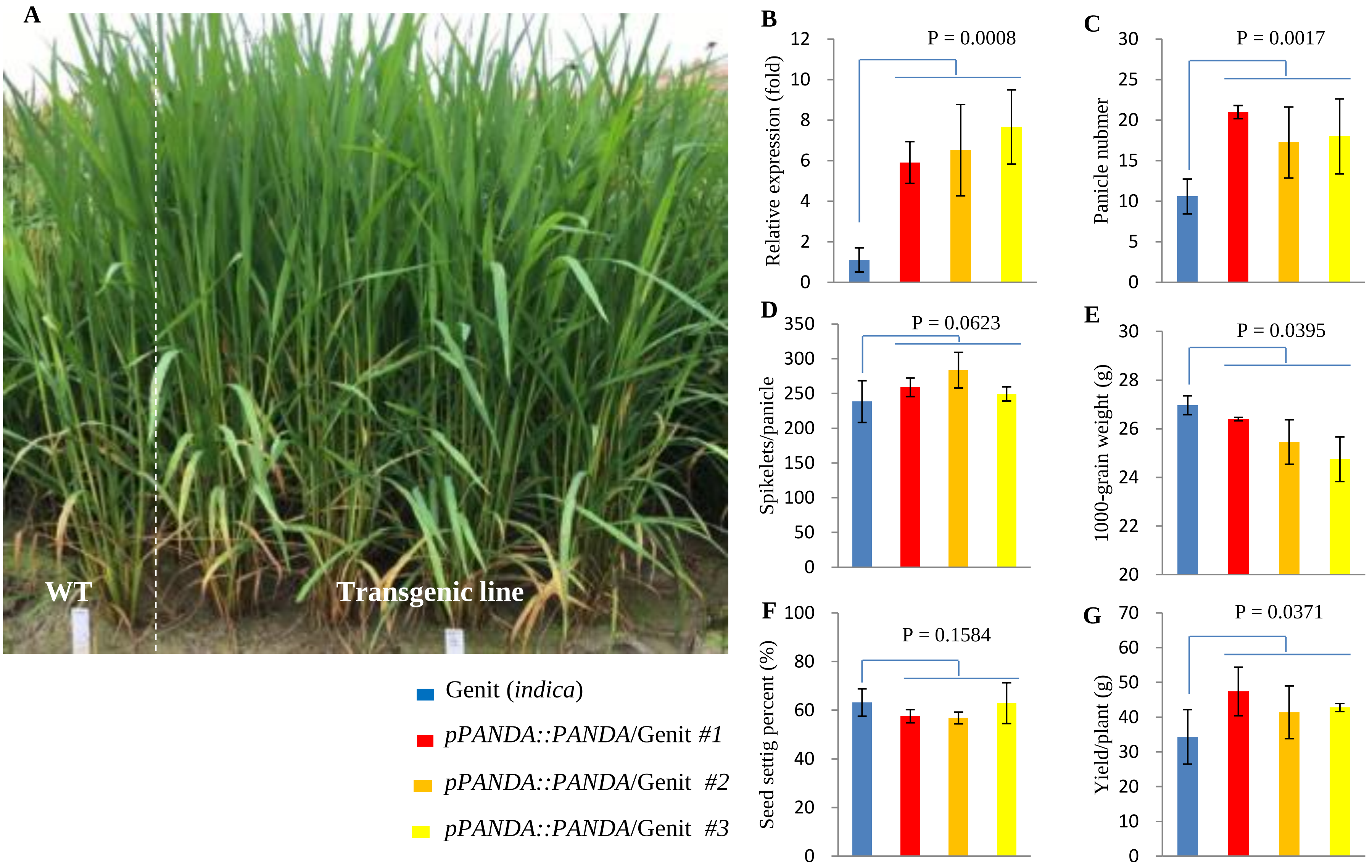
A
WT
Transgenic line
B
P = 0.0008
Relative expression (fold)
0
2
4
6
8
10
12
C
P = 0.0017
Panicle nubmer
0
5
10
15
20
25
30
D
P = 0.0623
Spikelets/panicle
0
50
100
150
200
250
300
350
E
P = 0.0395
1000-grain weight (g)
20
22
24
26
28
30
F
P = 0.1584
Seed settig percent (%)
0
20
40
60
80
100
G
P = 0.0371
Yield/plant (g)
0
10
20
30
40
50
60
70
Genit (*indica*)
pPANDA::PANDA/Genit *#1*
pPANDA::PANDA/Genit *#2*
pPANDA::PANDA/Genit *#3*

Fig. S7

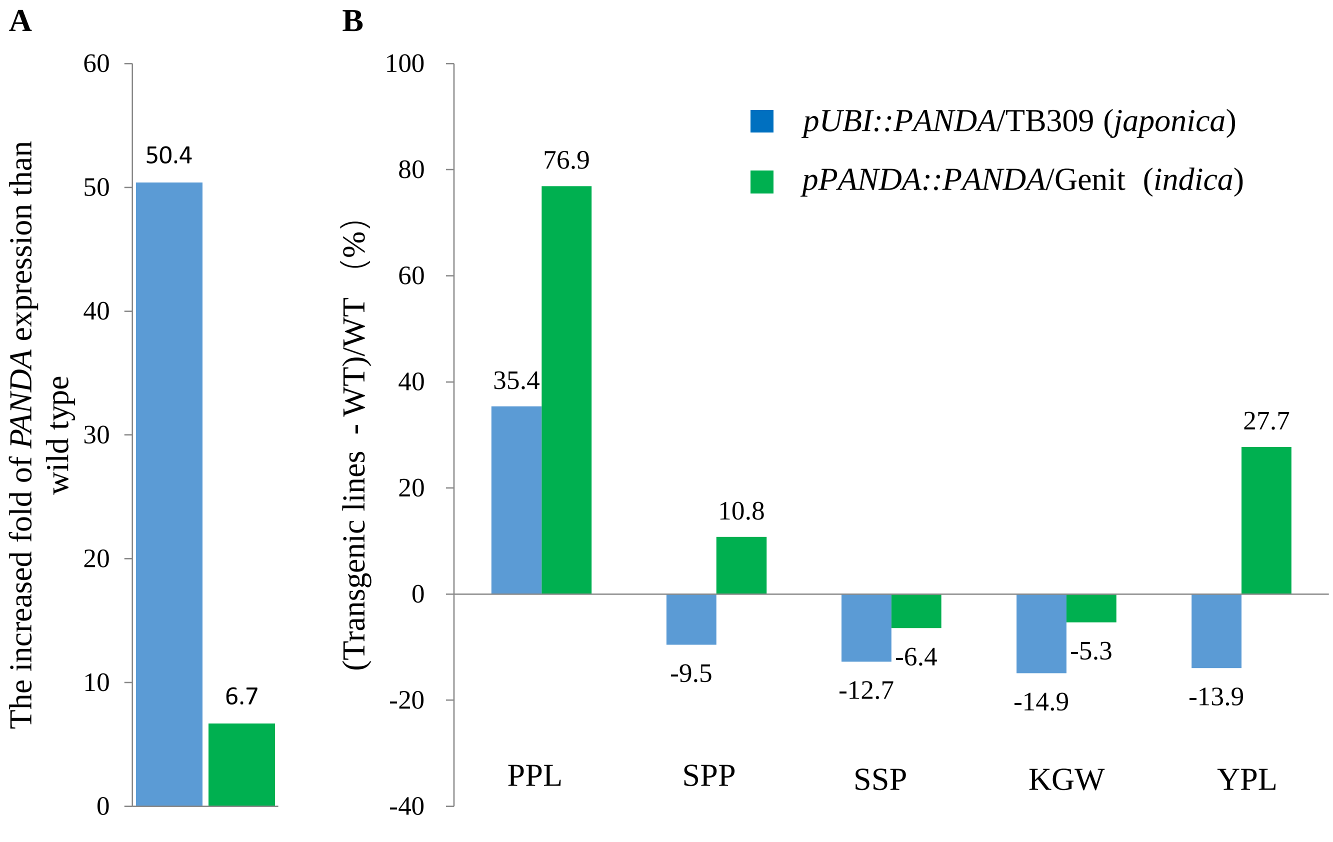

A
B
60
50
40
30
20
10
0
50.4
6.7
The increased fold of *PANDA* expression than wild type
100
80
60
40
20
0
-20
-40
(Transgenic lines - WT)/WT (%)
pUBI::PANDA/TB309 (*japonica*)
pPANDA::PANDA/Genit (*indica*)
35.4
76.9
-9.5
10.8
-12.7
-6.4
-14.9
-5.3
-13.9
27.7
PPL
SPP
SSP
KGW
YPL

Fig. S8

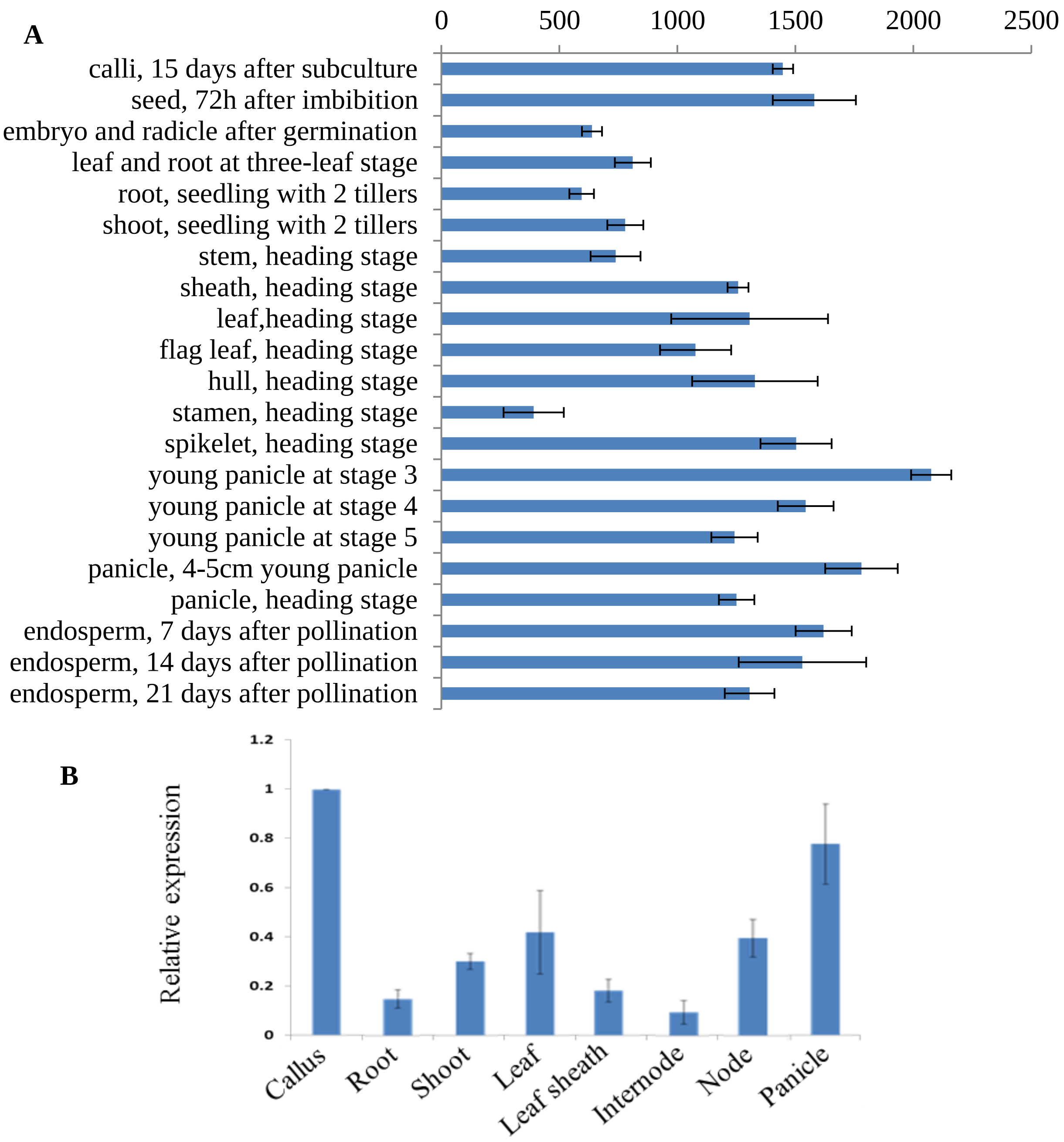
A
0
500
1000
1500
2000
2500
calli, 15 days after subculture
seed, 72h after imbibition
embryo and radicle after germination
leaf and root at three-leaf stage
root, seedling with 2 tillers
shoot, seedling with 2 tillers
stem, heading stage
sheath, heading stage
leaf,heading stage
flag leaf, heading stage
hull, heading stage
stamen, heading stage
spikelet, heading stage
young panicle at stage 3
young panicle at stage 4
young panicle at stage 5
panicle, 4-5cm young panicle
panicle, heading stage
endosperm, 7 days after pollination
endosperm, 14 days after pollination
endosperm, 21 days after pollination
B
Relative expression
1.2
1
0.8
0.6
0.4
0.2
0
Callus
Root
Shoot
Leaf
Leaf sheath
Internode
Node
Panicle

Fig. S9

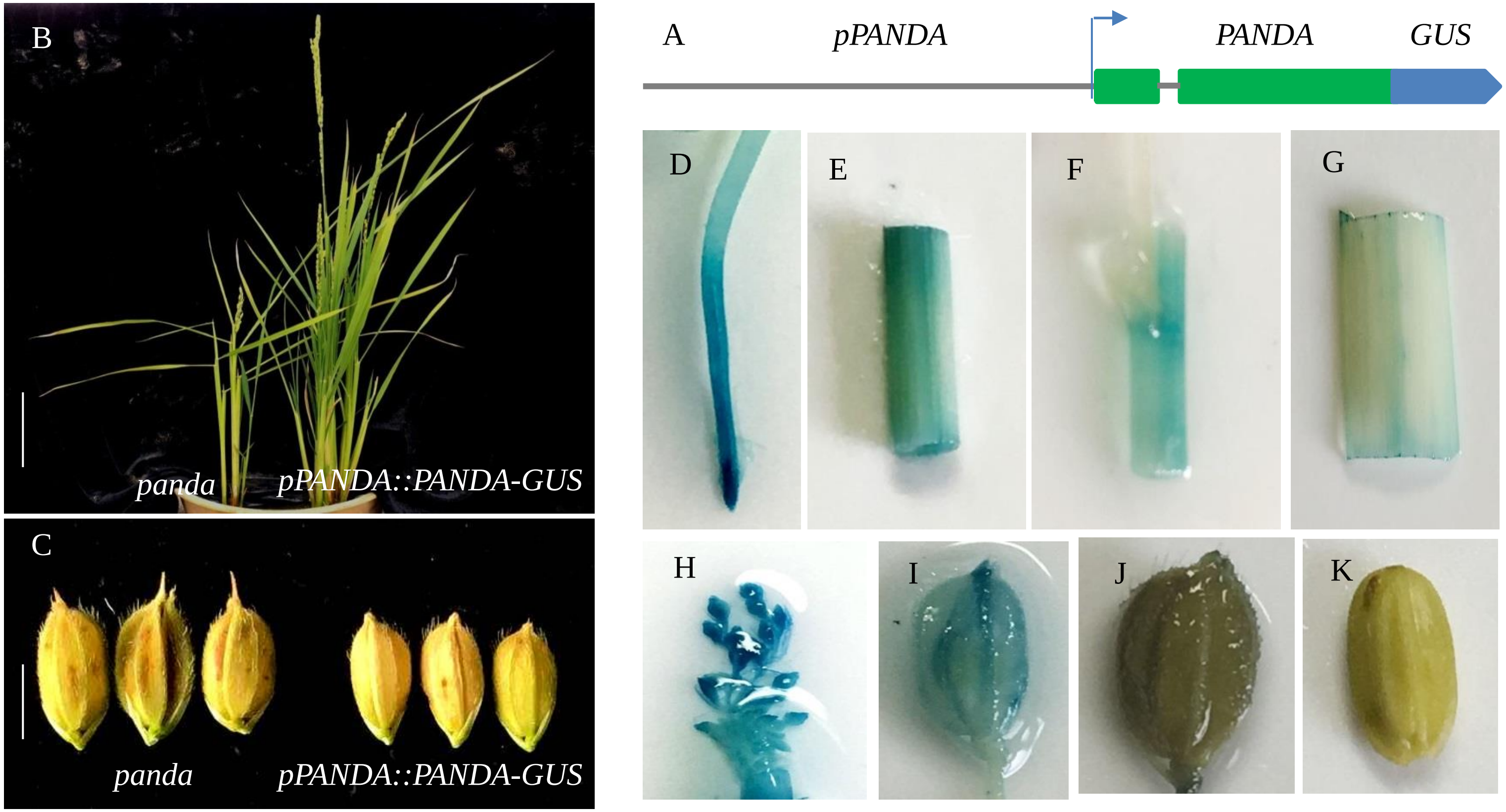
B
panda
pPANDA::PANDA-GUS
C
panda
pPANDA::PANDA-GUS
A
pPANDA
PANDA
GUS
D
E
F
G
H
I
J
K

Fig. S10

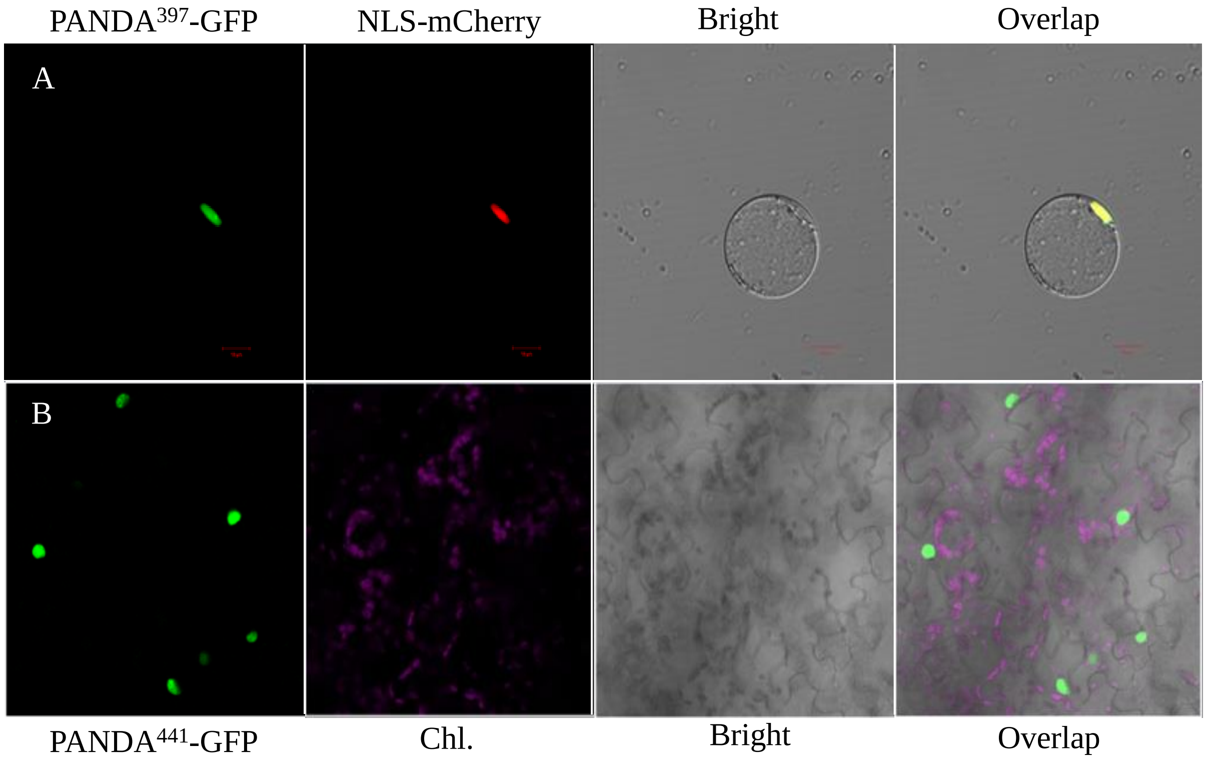
PANDA397-GFP
NLS-mCherry
Bright
Overlap
A
B
PANDA441-GFP
Chl.
Bright
Overlap

Fig. S11

A

Query ID	Subject ID	Cover %	Identify %	E value
PANDA	ALP1_Arabidopsis	83.0%	64.4%	2.00E-180
PANDA	Harbinger-4_CMy_1p	55.0%	36.1%	3.00E-47
PANDA	Harbinger-2_AMi_1p	62.0%	31.1%	3.00E-34
PANDA	Ping (ORF-B)	57.0%	23.4%	4.00E-09

D

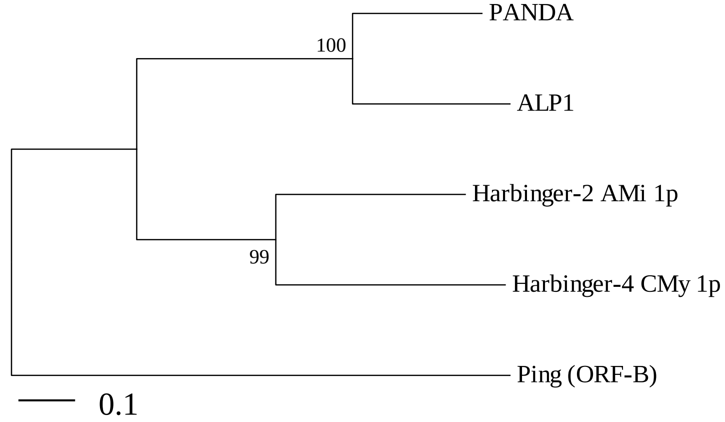

E *Ping*

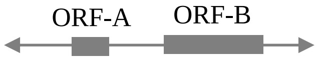

B

```
PING                IFRRRFRMYRPLFLRIVDALGQWSD-YFTQRVDAAGRQGLSPLQKCTAAIRQLATGSGAD  144
PANDA               AFRYFFRTSRRTFDYICSIVREDLISRPPSGLINIEGRLLSVEKQVAIAMRRLASGDSQV  168
Harbinger-2_AMi_1p  RWTQTFRMSRGTFMHLVAQVAPNIERQ-----DTVMRPALTPEKRVAIAIMRLATPASLR  187
                     :   **  *  *  :   :                   *:  :: : *: :**:  .

PING                ELDEYLKIGETTAMDAMKNFVKGIREVFGERYLRRPTVEDTERLLELGERRGFPGMFGSI  204
PANDA               SVGAAFGVGQSTVSQVTWRFIESMEERARHHLVWPGQERMEQIKARFEAESGLPNCCGAI  228
Harbinger-2_AMi_1p  YVAHSFGVGKSTAGQAVLDVCGAIQDTLADRVIRLAD--PGEVVAGF-GALGFPNCVGAL  244
                     :   : :*::*. :.   .  .:.:   .: :        :    :    *:*.  *::

PING                DCMHWQWERCPTAWKGQFTRGDQKVPTLILEAVASHDLWIWHAFFGVAGSNNDINVLSRS  264
PANDA               DATHIIMTLPAVESSEDWC-DPAKNYSMFLQGIVDDEMRFIDIVTGWPGSMMFSRLLKCS  287
Harbinger-2_AMi_1p  DGTHIPILRPPG-GGRQYI-NRRGSNSVVLEAVVDHRGRFTHIYTGWAGSARDAHVLRHS  302
                    *  *            ::  .     ::.*:.:...   : .   *  **    .:*  *

PING                TVFINEL-KGQAPRVQYMVNGNQYNEGYFLADGIYPEWKVFAKSYRLPIT-EKEKLYAQH  322
PANDA               GFFKHCDAGTRLDGPVMVSAENGEIREYIVGNNCYPLLPWLMTPYEGESLSAPMASFNAR  347
Harbinger-2_AMi_1p  PLPGLMQARTFAPGIQDLTIGDVSIPPVIVADAAYPLLPWLMRPFPGPLD-ARQQRYNEA  361
                     .               :   :      ::.:   **   :   :           :

PING                QEGARKDIERAFGVLQRRFCILKRPARLYDRGVLRDVVLGCIILHNMIVEDEKEARL---  379
PANDA               QKAARTLGPRALSRLKGSWRILNKVMWRPDKNKLPSIILVCCLLHNIIIDCEDELLPDVQ  407
Harbinger-2_AMi_1p  LARCRCTVEQAFGHLKGRWRSLATRLE-AAPQHIPCIITAACVLHNVCESRGEVFEPQ--  418
                       .*    :*:. *:  :  *           :  ::  . :***:  .  .
```

C

```
PANDA               #1   GGAGAAGCAG GTGGCGATTG CCATGAGGAG GCTGG:C:GT
Harbinger-2_AMi     #1   GGAGAAGCGT GTGGCCATAG CCATCATGAG GCTGGCCACA
                                 **      *  *      * *         * ***

PANDA               #41  CGGGCGATTC GCAGGTGTCG GTGGGGGCGG CTTTTGGTGT
Harbinger-2_AMi     #41  CCTGCCAGCC TCAGGTAT:: GTGGCCCACT CCTTTGGGGT
                           **  * **  *     * **     ******  *     *

PANDA               #81  CGGGCAGTCC ACCGTCTCG: CAGG:TGACT TGGAGGTTCA
Harbinger-2_AMi     #81  GGGGAAGTCC ACCG:CTGGC CAGGCTGTCT TGGATG:T:A
                         *   *          *  * *     *  *       * * *

PANDA               #121 T:CGAGTCGA TGGAAGAGCG GGCTCGGCAT CATCTGGTGT
Harbinger-2_AMi     #121 TGTGGGGCCA TCCAGGA:CA CCCT:AGC:: CGAC:CGAGT
                          ** * * *   ** *  * * **  **  **  ** ** *

PANDA               #161 GGCCCGGGCA GG:AGAGGAT GGAGCAGATC AAGGCGAGGT
Harbinger-2_AMi     #161 CATCC:GGCT GGCCGACCCA GG:GGAGGTG GTGGCAGGGT
                         ***  *   *   **  ****   * *  * * **   **

PANDA               #201 TCGAGGCCGA GTCCGGTCTG CCGAA:T:TG TTGCGGCGCC
Harbinger-2_AMi     #201 TTGGGGCC:: CTTGGGT:TC CCCAACTGTG TAGGGGCG:C
                          * *    ** * **   * *   *  * *    * *    *

PANDA               #241 ATCGATGCGA CCCACAT
Harbinger-2_AMi     #241 :TCGATGGGA CCCACAT
                         *      *
```

Fig. S12

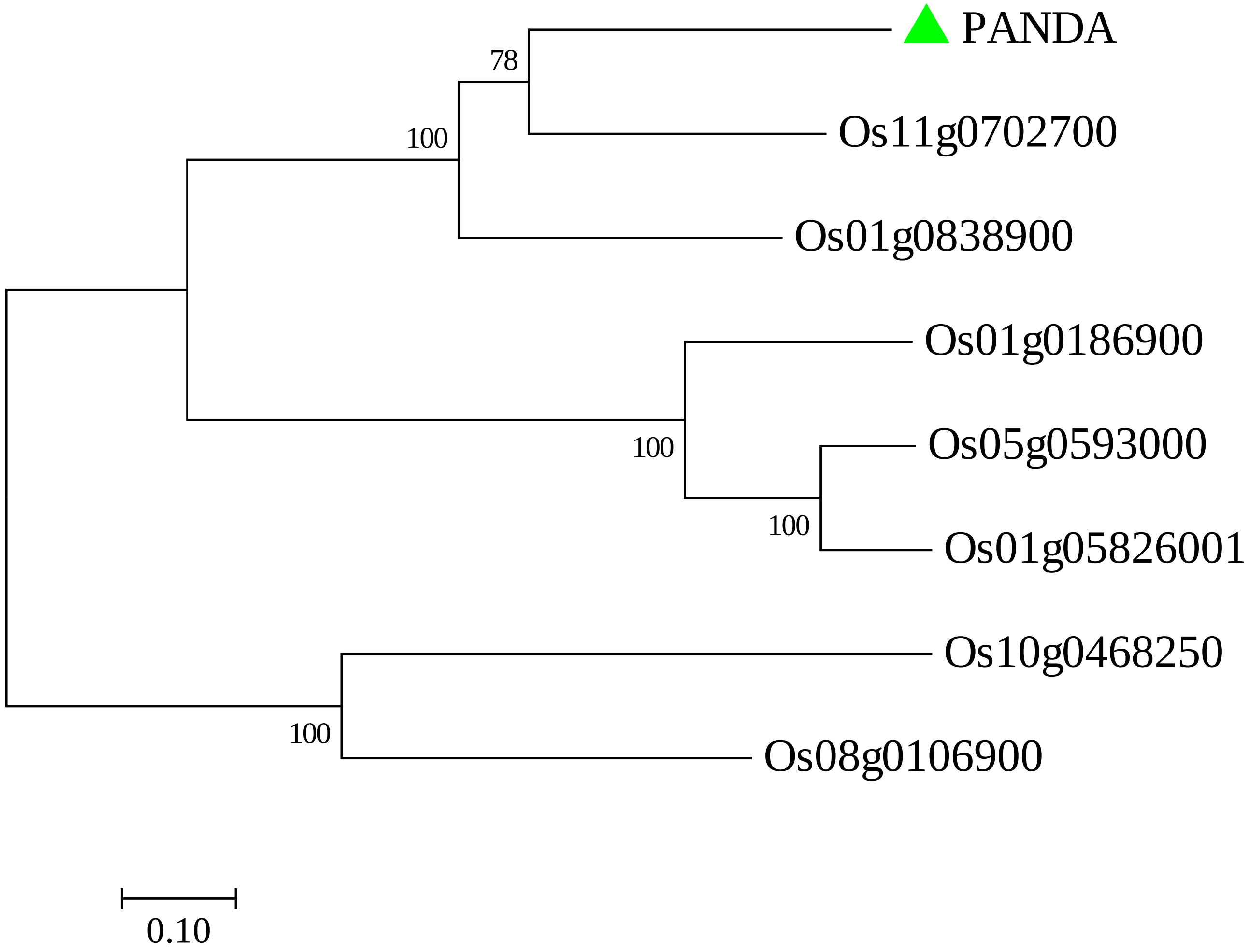
PANDA
Os11g0702700
Os01g0838900
Os01g0186900
Os05g0593000
Os01g05826001
Os10g0468250
Os08g0106900
78
100
100
100
100
0.10

Fig. S13

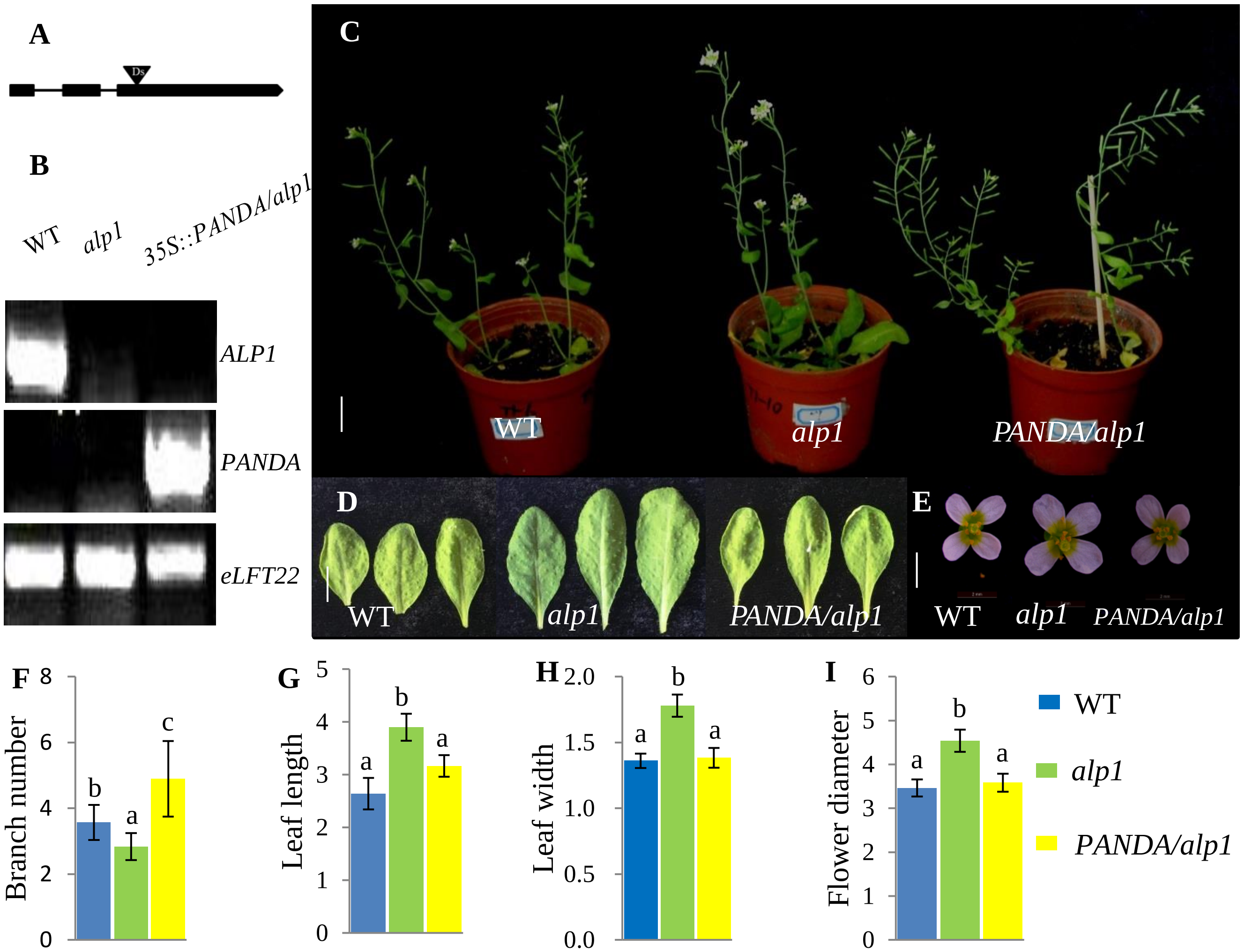
A
Ds
B
WT
alp1
35S::PANDA/alp1
ALP1
PANDA
eLFT22
C
WT
alp1
PANDA/alp1
D
WT
alp1
PANDA/alp1
E
WT
alp1
PANDA/alp1
F
Branch number
0
2
4
6
8
b
a
c
G
Leaf length
0
1
2
3
4
5
a
b
a
H
Leaf width
0.0
0.5
1.0
1.5
2.0
a
b
a
I
Flower diameter
0
1
2
3
4
5
6
a
b
a
WT
alp1
PANDA/alp1

Fig. S14

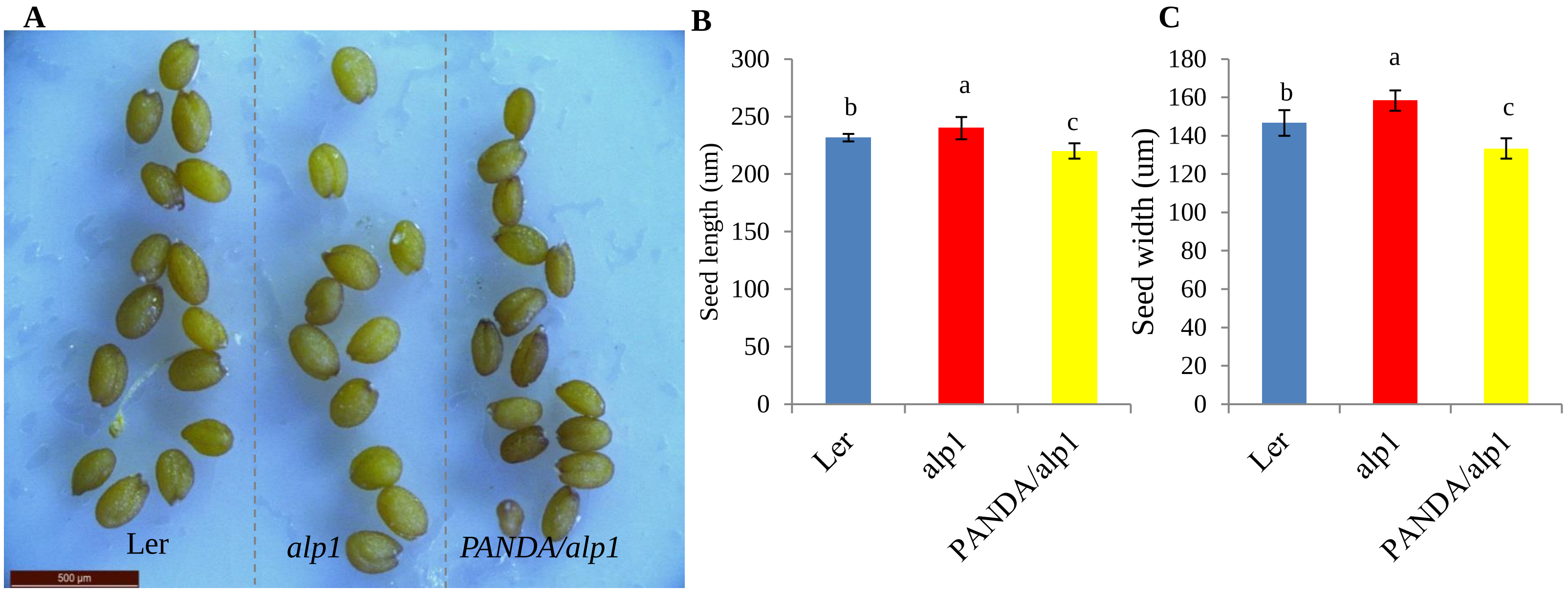
A
Ler
alp1
PANDA/alp1
500 µm
B
300
250
200
150
100
50
0
Seed length (um)
b
a
c
Ler
alp1
PANDA/alp1
C
180
160
140
120
100
80
60
40
20
0
Seed width (um)
b
a
c
Ler
alp1
PANDA/alp1

Fig. S15

A

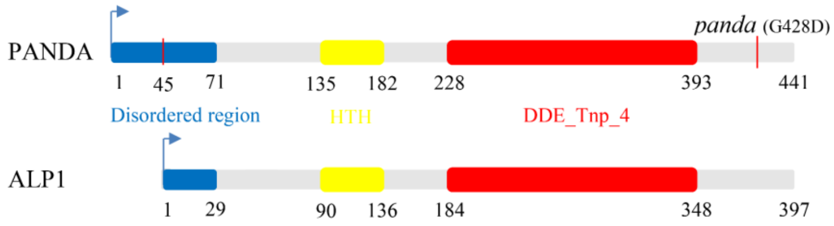

B

```
ALP1                                                 MAPVKQKKKNKKKPLD   16
PANDA     MEKKTKKKNPSKRGRKRGGRGEGREKKVEEISSSSSSRGRGRRRMAPVKKSKKGKRKSKD   60
                                                 *****:.**.*:*   *

ALP1      KAKKLAKNKEKKRVNAVPLDPEAIDCDWWDTFWLRNSS--PSVPSDEDYAFKHFFRASKT   74
PANDA     SGK-LKIVKYGGGAPPLPPELRGLDTEWWYTFLHKHSELGLSAPSDEGEAFRYFFRTSRR   119
          ..* *   *    .   :* : ..:* :** **  ::*.   *.****. **::***:*:

ALP1      TFSYICSLVREDLISRPPSGLINIEGRLLSVEKQVAIALRRLASGDSQVSVGAAFGVGQS   134
PANDA     TFDYICSIVREDLISRPPSGLINIEGRLLSVEKQVAIAMRRLASGDSQVSVGAAFGVGQS   179
          **.****:******************************:*********************

ALP1      TVSQVTWRFIEALEERAKHHLRWPDSDRIEEIKSKFEEMYGLPNCCGAIDTTHIIMTLPA   194
PANDA     TVSQVTWRFIESMEERARHHLVWPGQERMEQIKARFEAESGLPNCCGAIDATHIIMTLPA   239
          ***********::****:*** **..:*:*:**::**   **********:*********

ALP1      VQASDDWCDQEKNYSMFLQGVFDHEMRFLNMVTGWPGGMTVSKLLKFSGFFKLCENAQIL   254
PANDA     VESSEDWCDPAKNYSMFLQGIVDDEMRFIDIVTGWPGSMMFSRLLKCSGFFKHCDAGTRL   299
          *::*:****  *********:.*.****:::******.* .*:*** ***** *: .   *

ALP1      DGNPKTLSQGAQIREYVVGGISYPLLPWLITPHDSDHPSDSMVAFNERHEKVRSVAATAF   314
PANDA     DGPVMVSAENGEIREYIVGNNCYPLLPWLMTPYEGESLSAPMASFNARQKAARTLGPRAL   359
          **   . ::..:****:**. .*******:**::.:  *  *.:** *:: .*::.  *:

ALP1      QQLKGSWRILSKVMWRPDRRKLPSIILVCCLLHNIIIDCGDYLQEDVPLSGHHDSGYADR   374
PANDA     SRLKGSWRILNKVMWRPDKNKLPSIILVCCLLHNIIIDCEDELLPDVQLPDHHDTGYSEE   419
          .:*********.*******:.******************* *  ** * .***:**:.

ALP1      YCKQTEPLGSELRGCLTEHLLR     396
PANDA     KCEQVDPNGKIMRDVITGYLQI     441
           . .  . .   .   . .
```

Fig. S16

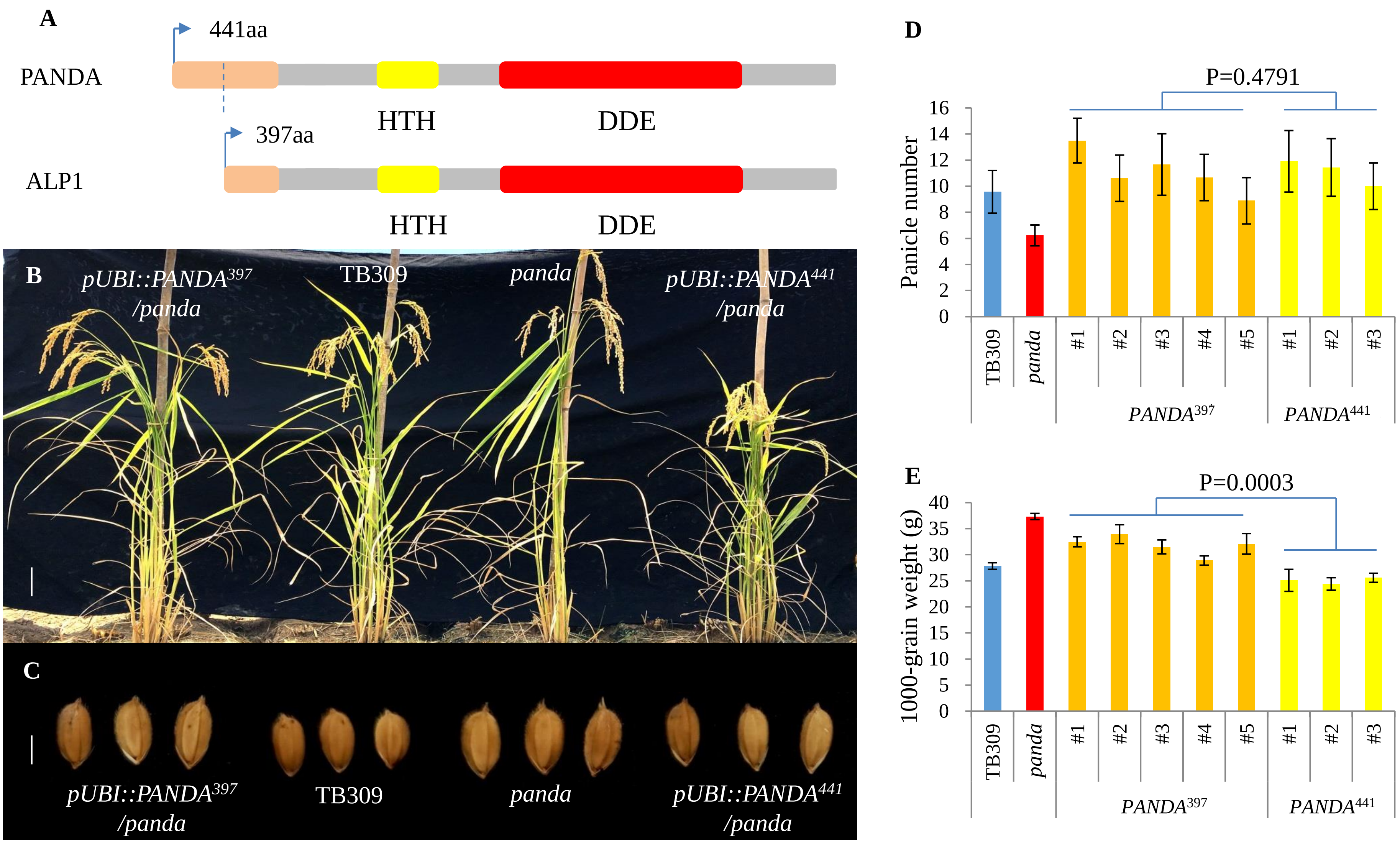
A
441aa
PANDA
397aa
HTH
DDE
ALP1
HTH
DDE
B
pUBI::PANDA397 /panda
TB309
panda
pUBI::PANDA441 /panda
C
pUBI::PANDA397 /panda
TB309
panda
pUBI::PANDA441 /panda
D
P=0.4791
Panicle number
16
14
12
10
8
6
4
2
0
TB309
panda
#1
#2
#3
#4
#5
#1
#2
#3
PANDA397
PANDA441
E
P=0.0003
1000-grain weight (g)
40
35
30
25
20
15
10
5
0
TB309
panda
#1
#2
#3
#4
#5
#1
#2
#3
PANDA397
PANDA441

Fig. S17

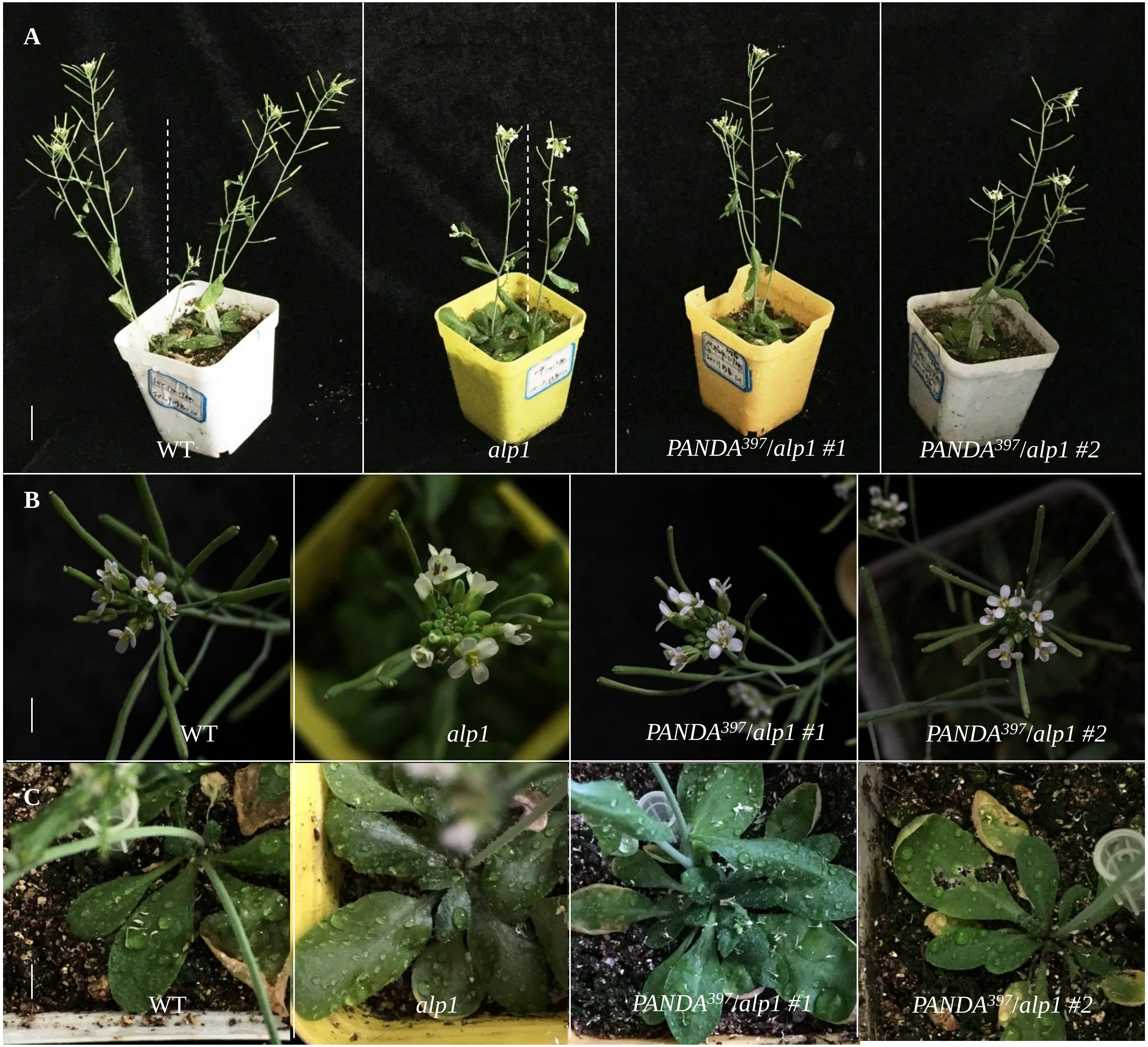
A
WT
alp1
PANDA397/alp1 #1
PANDA397/alp1 #2
B
WT
alp1
PANDA397/alp1 #1
PANDA397/alp1 #2
C
WT
alp1
PANDA397/alp1 #1
PANDA397/alp1 #2

Fig. S18

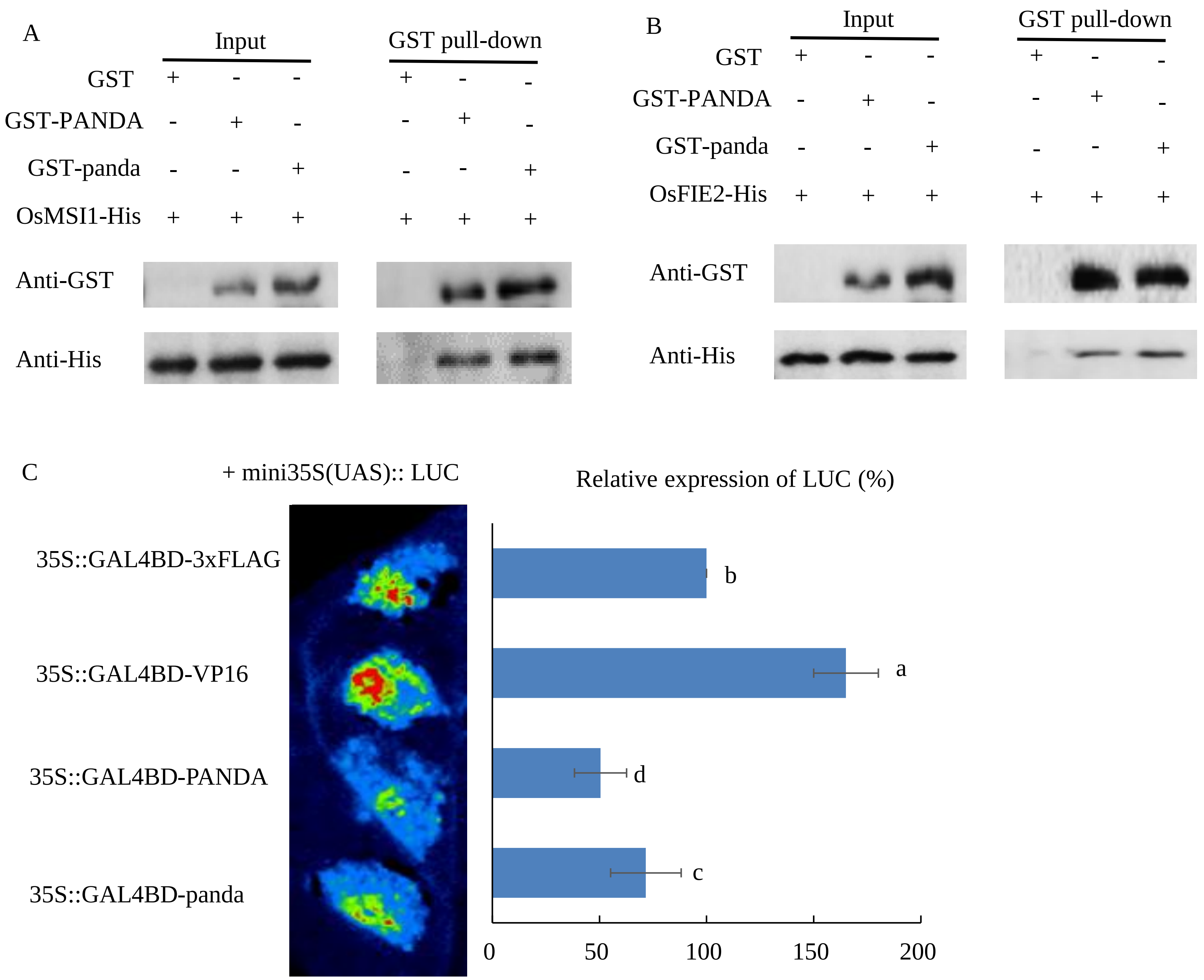
A
Input
GST pull-down
GST
GST-PANDA
GST-panda
OsMSI1-His
Anti-GST
Anti-His
B
Input
GST pull-down
GST
GST-PANDA
GST-panda
OsFIE2-His
Anti-GST
Anti-His
C
+ mini35S(UAS):: LUC
Relative expression of LUC (%)
35S::GAL4BD-3xFLAG
35S::GAL4BD-VP16
35S::GAL4BD-PANDA
35S::GAL4BD-panda
b
a
d
c
0
50
100
150
200

Fig. S19

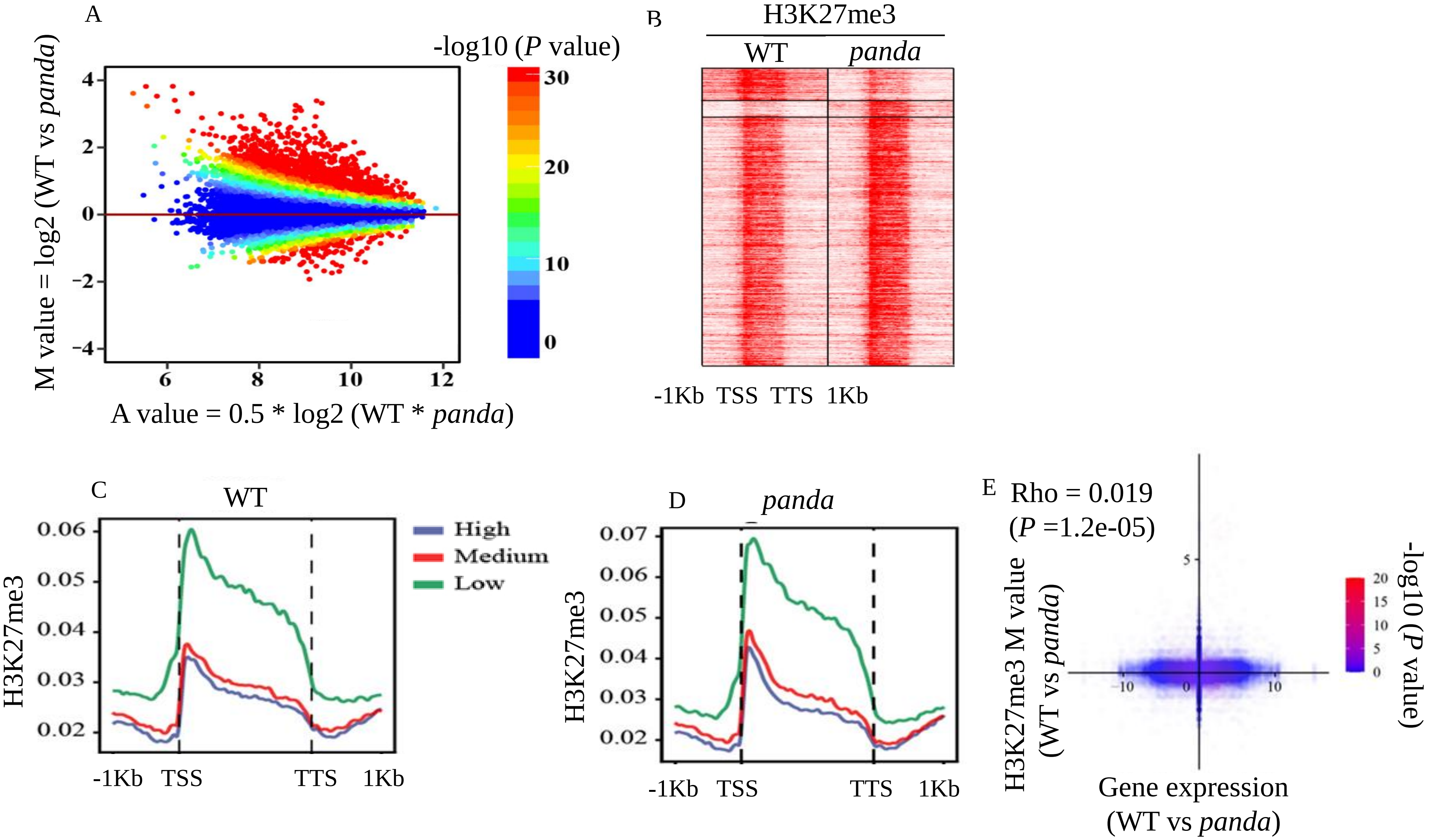
A
-log10 (P value)
30
20
10
0
4
2
0
−2
−4
6
8
10
12
M value = log2 (WT vs panda)
A value = 0.5 * log2 (WT * panda)
B
H3K27me3
WT
panda
-1Kb TSS TTS 1Kb
C
WT
High
Medium
Low
0.06
0.05
0.04
0.03
0.02
H3K27me3
-1Kb TSS TTS 1Kb
D
panda
0.07
0.06
0.05
0.04
0.03
0.02
H3K27me3
-1Kb TSS TTS 1Kb
E
Rho = 0.019
(P =1.2e-05)
5
-10
0
10
20
15
10
5
0
-log10 (P value)
H3K27me3 M value
(WT vs panda)
Gene expression
(WT vs panda)

Fig. S20

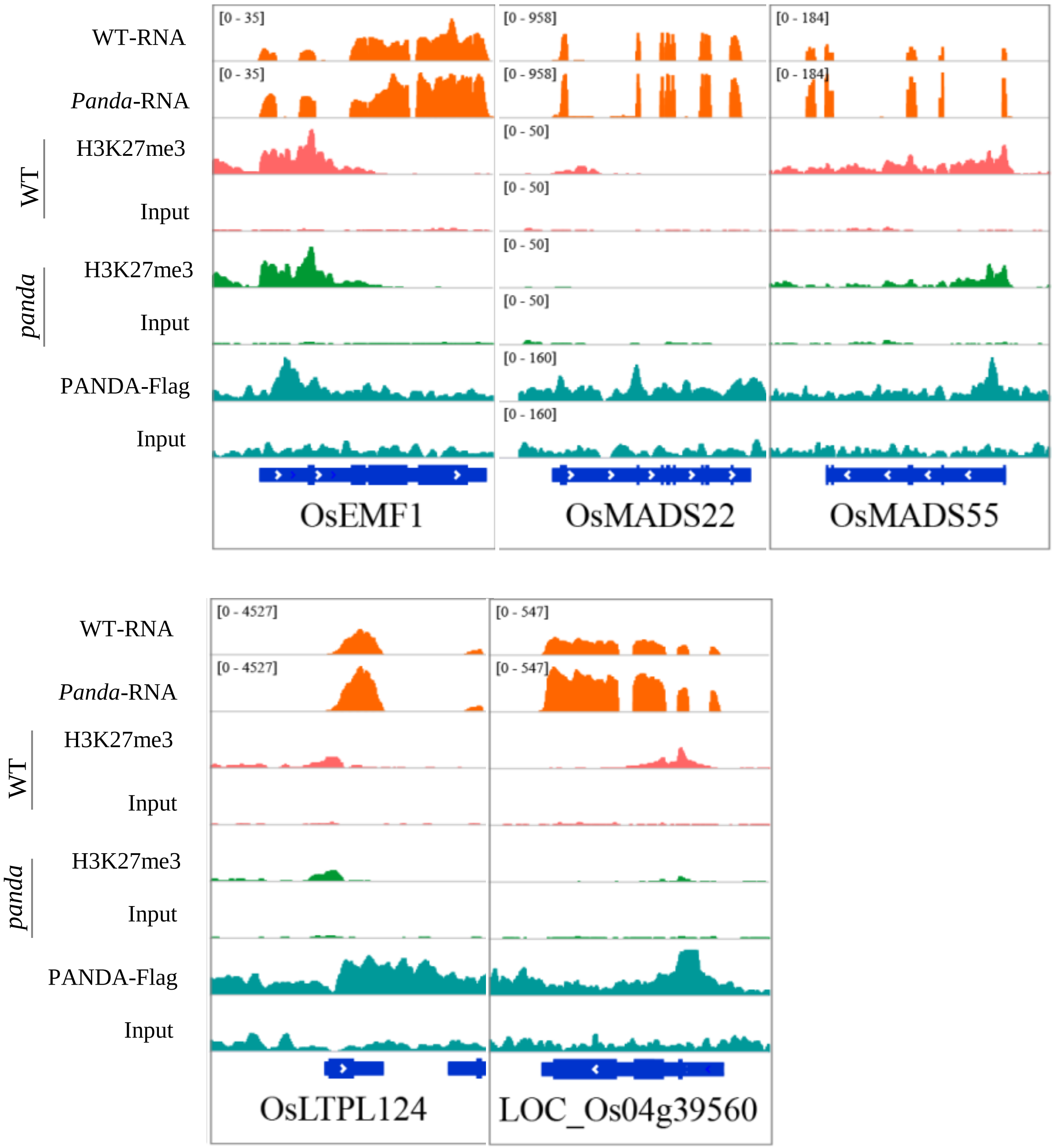
WT-RNA
Panda-RNA
H3K27me3
Input
WT
H3K27me3
Input
panda
PANDA-Flag
Input
[0 - 35]
[0 - 958]
[0 - 184]
[0 - 50]
[0 - 160]
OsEMF1
OsMADS22
OsMADS55
[0 - 4527]
[0 - 547]
OsLTPL124
LOC_Os04g39560

Fig. S21

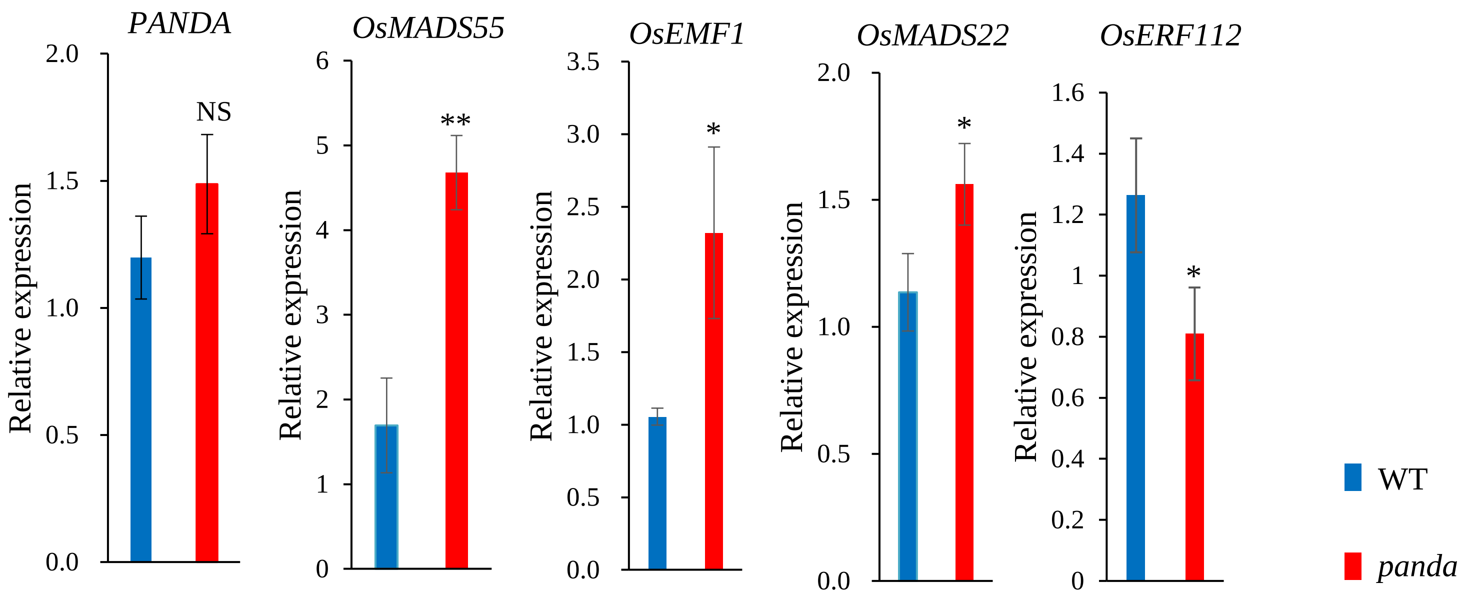
PANDA
OsMADS55
OsEMF1
OsMADS22
OsERF112
Relative expression
2.0
1.5
1.0
0.5
0.0
NS
6
5
4
3
2
1
0
**
3.5
3.0
2.5
*
*
1.6
1.4
1.2
1
0.8
0.6
0.4
0.2
WT
panda

Fig. S22

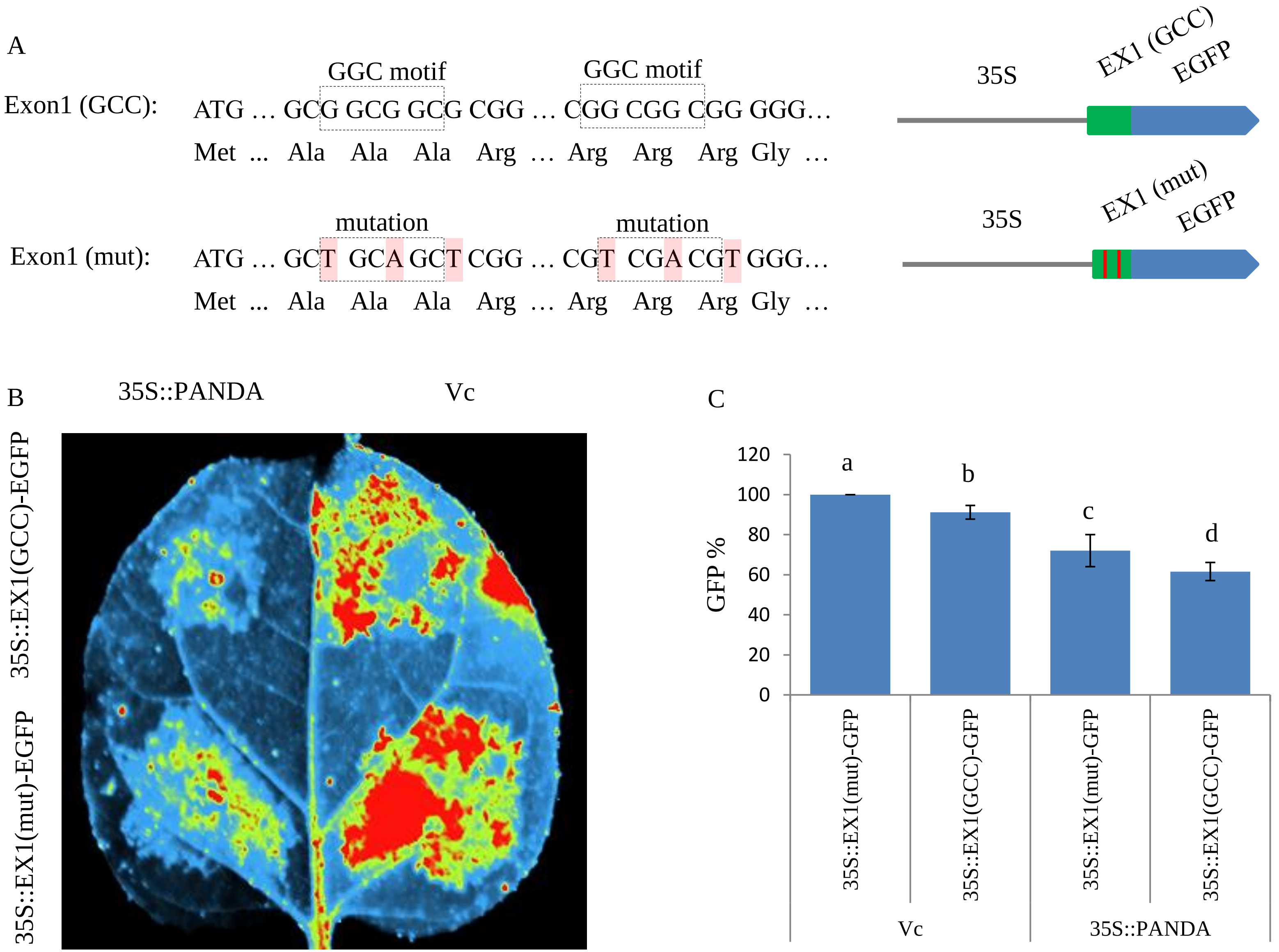
A
Exon1 (GCC):
GGC motif
GGC motif
ATG … GCG GCG GCG CGG … CGG CGG CGG GGG…
Met ... Ala Ala Ala Arg … Arg Arg Arg Gly …
35S
EX1 (GCC)
EGFP
Exon1 (mut):
mutation
mutation
ATG … GCT GCA GCT CGG … CGT CGA CGT GGG…
Met ... Ala Ala Ala Arg … Arg Arg Arg Gly …
35S
EX1 (mut)
EGFP
B
35S::PANDA
Vc
35S::EX1(GCC)-EGFP
35S::EX1(mut)-EGFP
C
GFP %
120
100
80
60
40
20
0
a
b
c
d
35S::EX1(mut)-GFP
35S::EX1(GCC)-GFP
35S::EX1(mut)-GFP
35S::EX1(GCC)-GFP
Vc
35S::PANDA

Fig. S23

PANDA	-	+	+	+	-	-	+
GCC motif- FITC	+	+	+	+	-	-	-
GCC motif	-	-	+	-	+	-	-
GCC motif with mutation	-	-	-	+	-	+	-

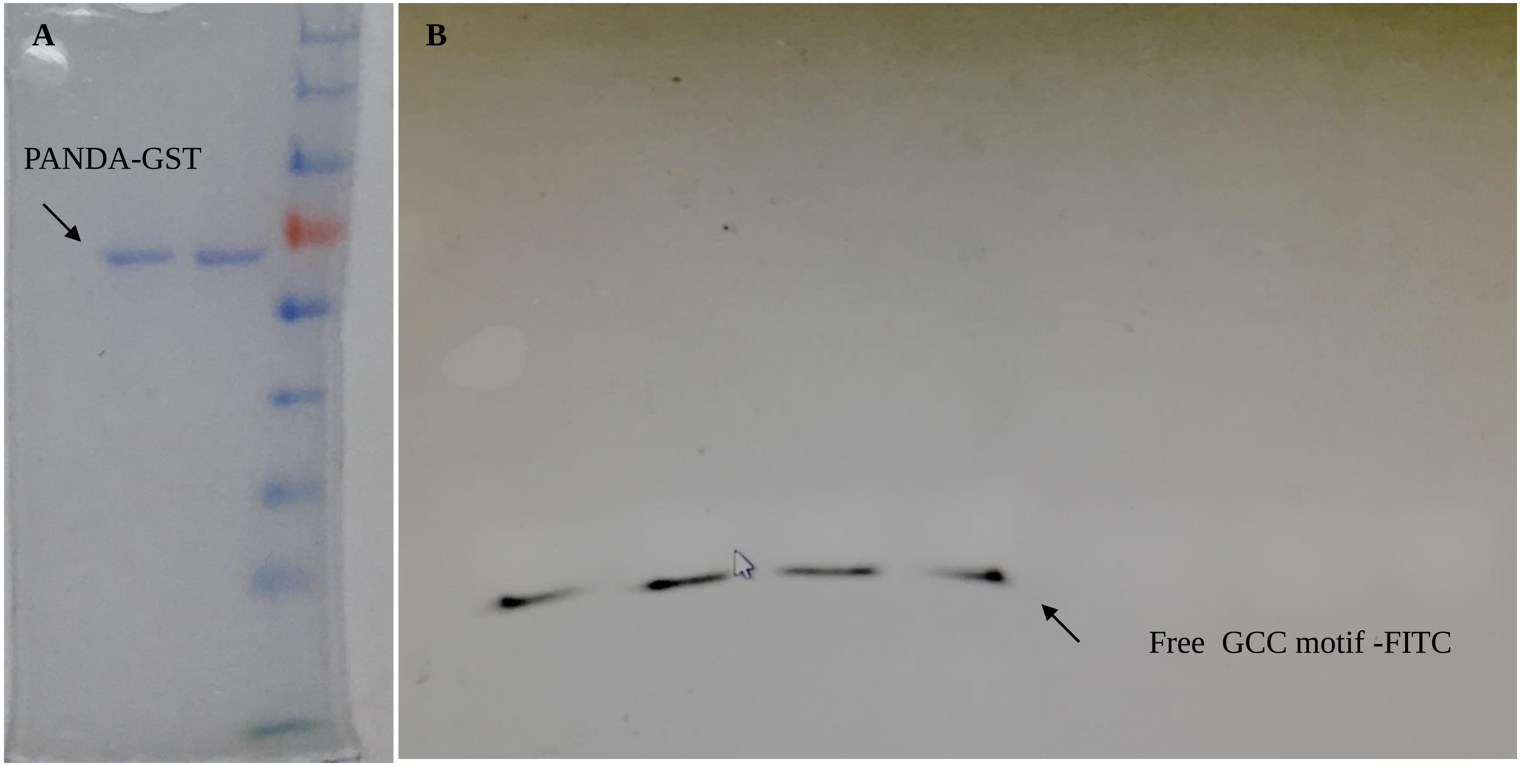

Fig. S24

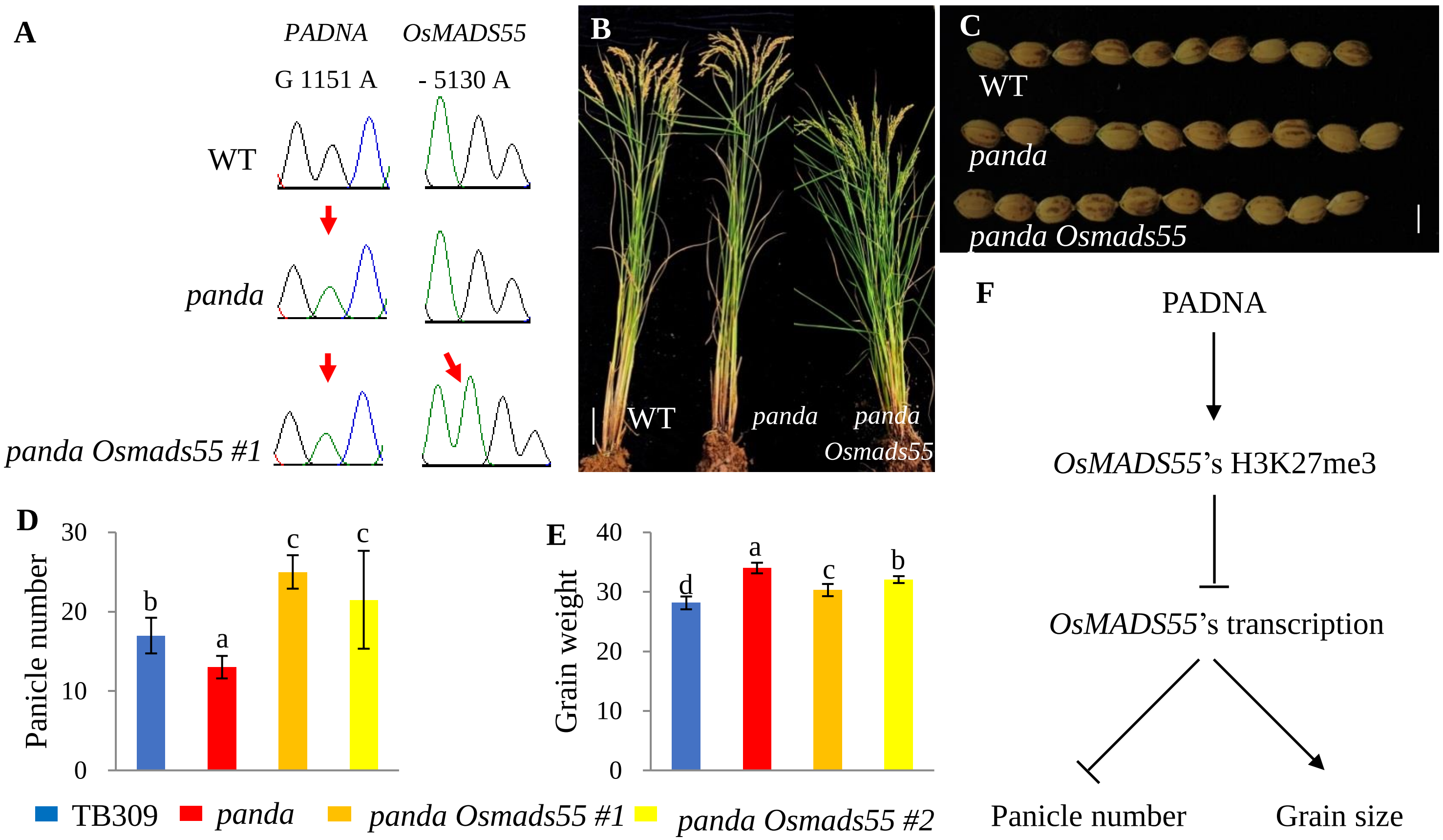
A
PADNA
OsMADS55
G 1151 A
- 5130 A
WT
panda
panda Osmads55 #1
B
WT
panda
panda Osmads55
C
WT
panda
panda Osmads55
D
Panicle number
30
20
10
0
b
a
c
c
E
Grain weight
40
30
20
10
0
d
a
c
b
TB309
panda
panda Osmads55 #1
panda Osmads55 #2
F
PADNA
OsMADS55's H3K27me3
OsMADS55's transcription
Panicle number
Grain size

Fig. S25

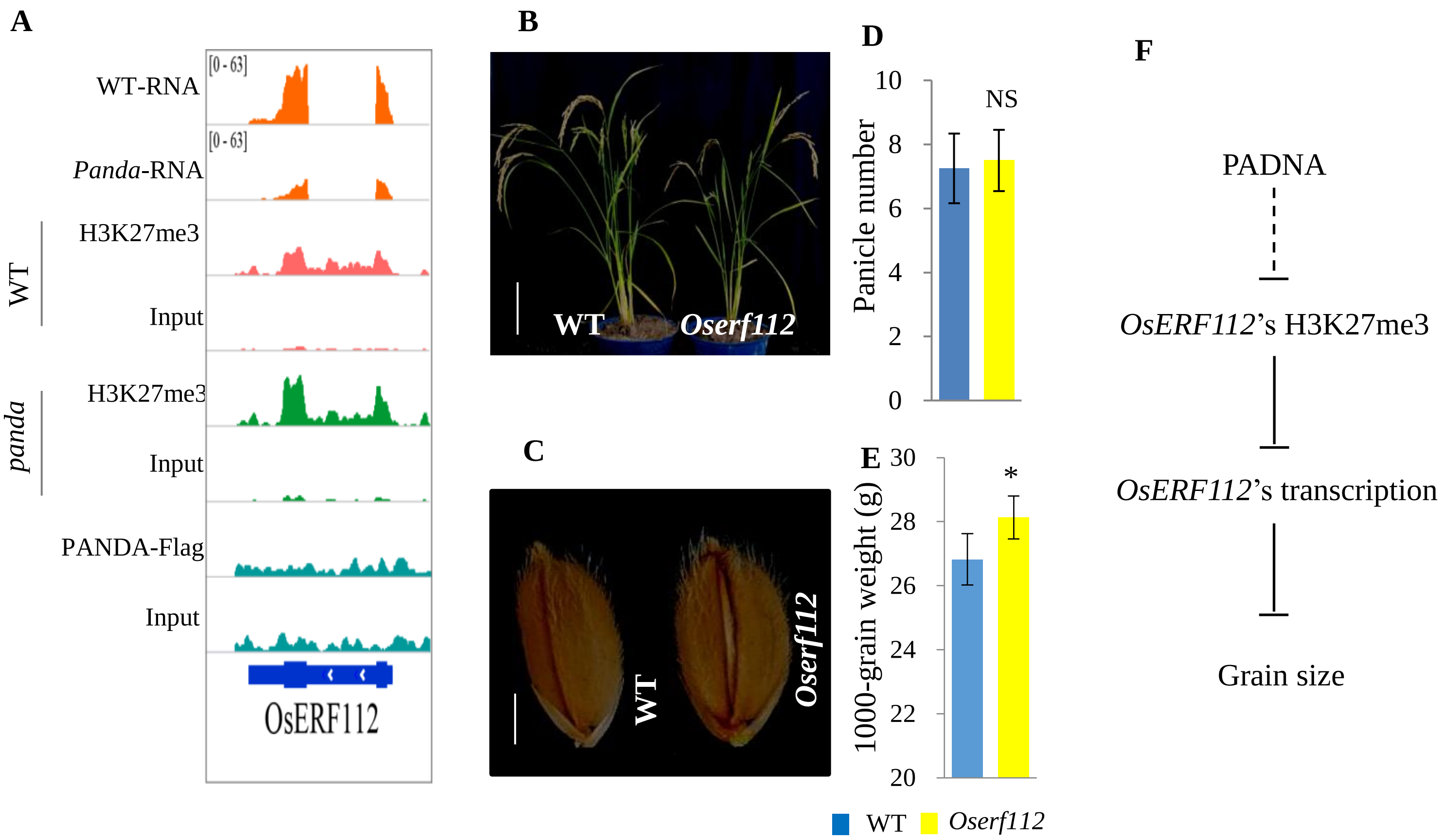
A
WT-RNA
[0 - 63]
Panda-RNA
[0 - 63]
WT
H3K27me3
Input
panda
H3K27me3
Input
PANDA-Flag
Input
OsERF112
B
WT
Oserf112
C
WT
Oserf112
D
Panicle number
NS
10
8
6
4
2
0
E
1000-grain weight (g)
*
30
28
26
24
22
20
WT
Oserf112
F
PADNA
OsERF112's H3K27me3
OsERF112's transcription
Grain size

Fig. S26
